# Supplementary material for: Lipase-Catalyzed Phospha-Michael Addition Reactions under Mild Conditions
Source: Molecules. 2022 Nov 12;27(22):7798. doi: 10.3390/molecules27227798 (PMC9698776; doi:10.3390/molecules27227798)
Supplement: Supplementary file 1 [file molecules-27-07798-s001.zip › molecules-1937412-supplementary.pdf]

Support information for

Article

Lipase-Catalyzed Phospha-Michael Addition Reactions under Mild Conditions

Yuelin Xu [1], Fengxi Li [1], Jinglin Ma [1], Jiapeng Li [1], Hanqing Xie [1], Chunyu Wang [2], Peng Chen [3,*] and Lei Wang [1,*]

[1] Key Laboratory of Molecular Enzymology and Engineering of Ministry of Education, School of Life Sciences, Jilin University, Changchun 130023, China
[2] State Key Laboratory of Supramolecular Structure and Materials, Jilin University, Changchun, 130023, China
[3] The Second Hospital of Jilin University Changchun, Jilin University, Changchun 130041, China
* Correspondence: c_p@jlu.edu.cn (P.C.); w_lei@jlu.edu.cn (L.W.)

Contents:

The Time Course 1

Reusability of Novozym 435 1

Data of Products 2

Spectra of Products 9

The Time Course

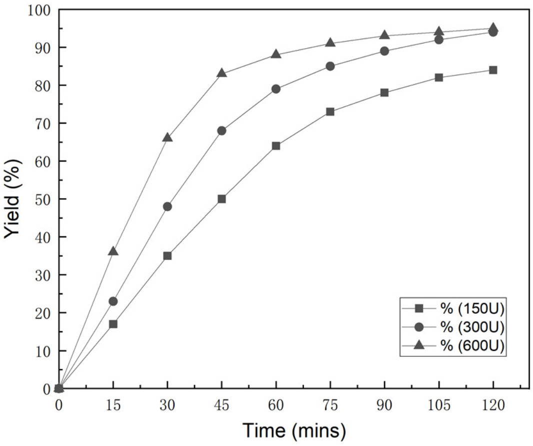

Figure S1. The time course of different dosage lipase-catalyzed phospha-Michael addition reaction. Reaction conditions: **1a** (0.5 mmol), **2** (0.5 mmol), Novozym 435, rt, and EtOH (1 mL).

Reusability of Novozym 435

Table S1. Reusability of Novozym 435 on the phospha-Michael addition reaction.

Cycle	Enzyme weight (mg)	Yield (%)
1	20.0	94
2	19.8	93
3	19.4	92
4	19.2	89
5	18.9	86
6	18.5	81
7	18.2	78
8	17.9	72

Reaction conditions: **1a** (0.5 mmol), **2** (0.5 mmol), Novozym 435 (300 U = 20mg), rt, and EtOH (1 mL).

Data of Products

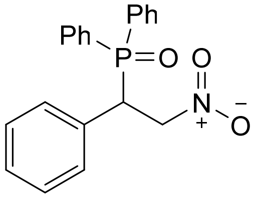

3a *((2-nitro-1-phenylethyl) diphenylphosphine oxide)*

White solid, yield: 165mg, 94%; ^{1}H NMR (400 MHz, DMSO-d_6) δ 8.18 – 8.10 (m, 2H), 7.78 – 7.70 (m, 2H), 7.68 – 7.62 (m, 3H), 7.41 (td, J = 7.2, 1.6 Hz, 3H), 7.37 – 7.31 (m, 2H), 7.23 – 7.12 (m, 3H), 5.15 – 4.97 (m, 2H), 4.91 – 4.81 (m, 1H). ^{13}C NMR (151 MHz, DMSO) δ 133.20, 132.62, 131.52, 131.21, 131.15, 131.06, 131.00, 130.62, 130.41, 129.81, 129.73, 128.98, 128.90, 128.83, 128.37, 76.47, 76.06, 44.44, 44.02. ^{31}P NMR (243 MHz, DMSO) δ 30.84.

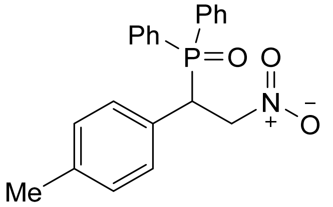

3b *((2-nitro-1-(p-tolyl) ethyl) diphenylphosphine oxide)*

White solid, yield: 173mg, 95%; ^{1}H NMR (400 MHz, Chloroform-d) δ 8.01 – 7.94 (m, 2H), 7.67 – 7.56 (m, 3H), 7.51 – 7.45 (m, 2H), 7.40 (dd, J = 7.2, 1.6 Hz, 1H), 7.30 (td, J = 6.4, 5.6, 3.6 Hz, 2H), 7.17 (dd, J = 8.0, 2.0 Hz, 2H), 7.02 (d, J = 7.6 Hz, 2H), 5.11 – 5.01 (m, 1H), 4.77 – 4.71 (m, 1H), 4.41 (s, 1H), 2.26 (d, J = 1.6 Hz, 3H). ^{13}C NMR (151 MHz, DMSO) δ 135.71, 130.35, 129.74, 128.85, 128.79, 128.69, 128.23, 128.09, 127.59, 127.40, 127.15, 126.91, 126.05, 125.97, 114.09, 74.98, 74.77, 74.56, 73.57, 73.53, 43.28, 42.86, 18.73. ^{31}P NMR (243 MHz, DMSO) δ 24.87.

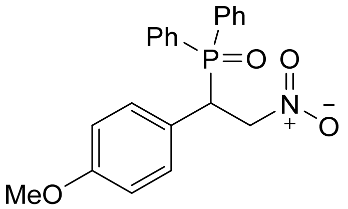

3c *((1-(4-methoxyphenyl)-2-nitroethyl) diphenylphosphine oxide)*

White solid, yield: 169mg, 89%; ^{1}H NMR (400 MHz, DMSO-d_6) δ 8.15 – 8.07 (m, 2H), 7.81 – 7.72 (m, 2H), 7.65 (dt, J = 6.0, 3.6 Hz, 3H), 7.45 – 7.32 (m, 5H), 6.80 – 6.74 (m, 2H), 5.06 – 4.90 (m, 2H), 4.84 – 4.77 (m, 1H), 3.67 (s, 3H). ^{13}C NMR (151 MHz, DMSO) δ 159.06, 133.08, 132.53, 131.18, 131.07, 130.28, 129.75, 129.67, 128.99, 128.91, 124.34, 114.20, 76.34, 55.38, 55.06, 43.58, 43.15. ^{31}P NMR (243 MHz, DMSO) δ 30.56.

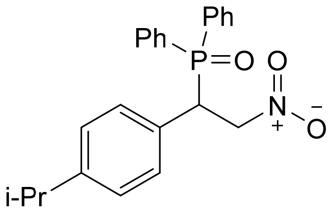

3d *((1-(4-isopropylphenyl)-2-nitroethyl) diphenylphosphine oxide)*

White solid, yield: 157mg, 80%; ^{1}H NMR (400 MHz, Chloroform-*d*) δ 8.01 (t, *J* = 9.2 Hz, 2H), 7.66 (dq, *J* = 14.0, 7.2 Hz, 3H), 7.43 (q, *J* = 7.6, 6.4 Hz, 3H), 7.31 (d, *J* = 2.8 Hz, 1H), 7.28 (s, 1H), 7.21 (d, *J* = 8.0 Hz, 2H), 7.09 (d, *J* = 7.2 Hz, 2H), 5.13 (s, 1H), 4.78 (d, *J* = 13.6 Hz, 1H), 4.45 (d, *J* = 10.8 Hz, 1H), 2.89 – 2.79 (m,1H), 1.20 (d, *J* = 6.9 Hz, 6H). ^{13}C NMR (151 MHz, $CDCl_3$) δ 149.03, 132.73, 132.53, 132.04, 131.31, 131.14, 130.76, 130.47, 129.81, 129.35, 129.27, 128.61, 128.28, 128.21, 126.84, 75.89, 75.71, 45.77, 45.33, 33.68, 23.82. ^{31}P NMR (243 MHz, $CDCl_3$) δ 30.26.

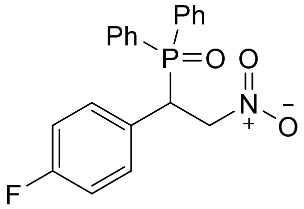

3e *((1-(4-fluorophenyl)-2-nitroethyl) diphenylphosphine oxide)*

White solid, yield: 160mg 87%; ^{1}H NMR (400 MHz, DMSO-d_6) δ 8.20 – 8.08 (m, 2H), 7.74 (ddd, *J* = 11.6, 6.8, 1.6 Hz, 2H), 7.69 – 7.63 (m, 3H), 7.51 – 7.33 (m, 5H), 7.06 (t, *J* = 8.8 Hz, 2H), 5.12 – 5.07 (m, 2H), 4.85 (s, 1H). ^{13}C NMR (151 MHz, DMSO) δ 139.74, 139.53, 133.09, 132.49, 131.88, 131.31, 131.25, 131.09, 131.04, 130.74, 130.39, 129.73, 129.65, 129.31, 128.93, 128.86, 115.70, 115.55, 76.24, 76.23, 43.56, 43.14. ^{31}P NMR (243 MHz, DMSO) δ 29.97.

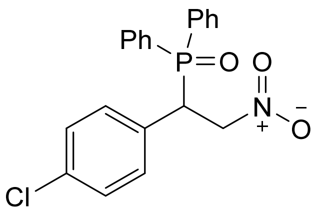

3f *((1-(4-chlorophenyl)-2-nitroethyl) diphenylphosphine oxide)*

White solid, yield: 169mg, 88%; ^{1}H NMR (400 MHz, Chloroform-*d*) δ 7.99 (dd, *J* = 11.2, 7.6 Hz, 2H), 7.70 – 7.59 (m, 3H), 7.53 – 7.41 (m, 3H), 7.34 (dt, *J* = 10.8, 5.2 Hz, 2H), 7.27 (dd, *J* = 8.4, 1.6 Hz, 2H), 7.21 (d, *J* = 8.4 Hz, 2H), 5.06 (d, *J* = 3.2 Hz, 1H), 4.78 – 4.70 (m, 1H), 4.47 – 4.38 (m, 1H). ^{13}C NMR (151 MHz, $CDCl_3$) δ 134.43, 132.92, 132.34, 131.15, 131.09, 130.95, 130.89, 130.74, 130.32, 130.13, 129.48, 129.40, 129.05, 128.59, 128.51, 75.69, 75.65, 45.46, 45.04. ^{31}P NMR (243 MHz, $CDCl_3$) δ 29.53.

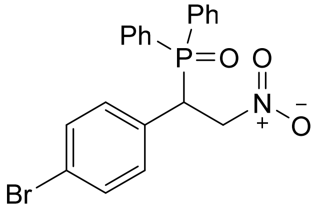

3g *((1-(4-bromophenyl)-2-nitroethyl) diphenylphosphine oxide)*

White solid, yield: 193mg, 90%; ^{1}H NMR (400 MHz, DMSO-d_6) δ 8.13 (td, J = 8.0, 3.6 Hz, 2H), 7.76 (dd, J = 11.6, 7.6 Hz, 2H), 7.69 – 7.62 (m, 3H), 7.48 – 7.35 (m, 7H), 5.13 – 5.05 (m, 2H), 4.90 – 4.80 (m, 1H). ^{13}C NMR (151 MHz, DMSO) δ 133.13, 132.71, 132.60, 132.19, 131.99, 131.67, 131.29, 131.10, 131.03, 130.65, 130.27, 129.75, 129.67, 128.94, 121.60, 76.00, 43.75, 43.33. ^{31}P NMR (243 MHz, DMSO) δ 29.69.

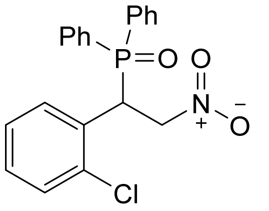

3h *((1-(2-chlorophenyl)-2-nitroethyl) diphenylphosphine oxide)*

White solid, yield: 161mg 84%; ^{1}H NMR (400 MHz, Chloroform-d) δ 8.10 (ddd, J = 11.2, 7.6, 1.6 Hz, 2H), 7.89 (d, J = 7.6 Hz, 1H), 7.74 – 7.64 (m, 3H), 7.49 – 7.39 (m, 3H), 7.34 (dt, J = 8.4, 4.0 Hz, 1H), 7.26 (dd, J = 7.6, 3.2 Hz, 2H), 7.19 (d, J = 4.0 Hz, 2H), 5.25 (dd, J = 9.2, 3.2 Hz, 1H), 5.20 – 5.11 (m, 1H), 4.79 (ddd, J = 13.6, 6.0, 3.2 Hz, 1H). ^{13}C NMR (151 MHz, $CDCl_3$) δ 134.95, 132.97, 132.26, 131.94, 131.30, 131.24, 130.95, 130.89, 130.19, 129.91, 129.71, 129.56, 129.47, 129.41, 128.42, 128.20, 128.12, 127.52, 75.39, 75.38, 41.14, 40.72. ^{31}P NMR (243 MHz, $CDCl_3$) δ 30.73.

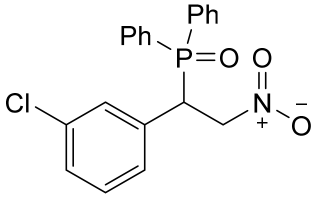

3i *((1-(3-chlorophenyl)-2-nitroethyl) diphenylphosphine oxide)*

White solid, yield: 154mg, 80%; ^{1}H NMR (400 MHz, Chloroform-d) δ 8.01 (dd, J = 11.2, 7.6 Hz, 2H), 7.67 (t, J = 9.2 Hz, 3H), 7.53 – 7.43 (m, 3H), 7.36 (dt, J = 8.8, 4.4 Hz, 2H), 7.28 – 7.16 (m, 4H), 5.15 – 5.03 (m, 1H), 4.80 – 4.73 (m, 1H), 4.46 – 4.36 (m, 1H). ^{13}C NMR (151 MHz, $CDCl_3$) δ 134.61, 133.83, 132.98, 132.41, 131.19, 131.13, 130.99, 130.93, 130.01, 129.65, 129.49, 129.42, 128.57, 128.49, 127.47, 75.45, 45.76, 45.34. ^{31}P NMR (243 MHz, $CDCl_3$) δ 29.82.

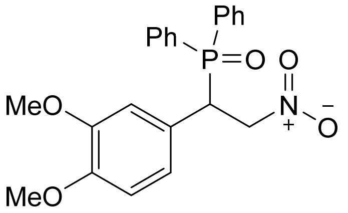

3j *(1-(3,4-dimethoxyphenyl)-2-nitroethyl) diphenylphosphine oxide*

White solid, yield: 154mg, 75%; ^{1}H NMR (400 MHz, Chloroform-*d*) δ 7.99 (dd, *J* = 11.2, 7.6 Hz, 2H), 7.70 – 7.59 (m, 3H), 7.53 – 7.41 (m, 3H), 7.34 (dt, *J* = 10.8, 5.2 Hz, 2H), 7.27 (dd, *J* = 8.4, 1.6 Hz, 2H), 7.21 (d, *J* = 8.4 Hz, 2H), 5.06 (d, *J* = 3.2 Hz, 1H), 4.78 – 4.70 (m, 1H), 4.47 – 4.38 (m, 1H), 3.88 (s, 3H), 3.77 (s, 3H). ^{13}C NMR (151 MHz, $CDCl_3$) δ 134.43, 132.92, 132.34, 131.15, 131.09, 130.95, 130.89, 130.74, 130.32, 130.13, 129.47, 129.40, 129.04, 128.59, 128.51, 122.45, 122.05, 122.00, 75.67, 55.45, 55.45, 45.46, 45.04. ^{31}P NMR (243 MHz, $CDCl_3$) δ 29.53.

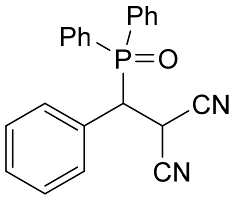

5a *(2-((diphenylphosphoryl)(phenyl)methyl) malononitrile)*

White solid, yield: 163mg, 92%; ^{1}H NMR (400 MHz, Chloroform-*d*) δ 8.05 – 7.97 (m, 2H), 7.72 – 7.60 (m, 3H), 7.56 – 7.49 (m, 2H), 7.45 – 7.39 (m, 3H), 7.31 (td, *J* = 5.2, 4.8, 3.2 Hz, 5H), 4.78 (t, *J* = 7.6 Hz, 1H), 4.09 (s, 1H). ^{13}C NMR (151 MHz, $CDCl_3$) δ 134.43, 132.92, 132.34, 131.15, 131.09, 130.95, 130.89, 130.74, 130.32, 130.13, 129.47, 129.40, 129.04, 128.59, 128.51, 111.14, 111.09, 111.05, 75.67, 45.46, 45.04, 24.31. ^{31}P NMR (243 MHz, $CDCl_3$) δ 29.53.

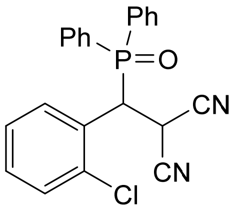

5b *(2-((2-chlorophenyl) (diphenylphosphoryl)methyl) malononitrile)*

White solid, yield: 173mg, 89%; ^{1}H NMR (400 MHz, DMSO-d_6) δ 8.20 – 8.12 (m, 2H), 7.99 (d, *J* = 8.0 Hz, 1H), 7.76 – 7.64 (m, 3H), 7.50 (dd, *J* = 11.6, 7.6 Hz, 2H), 7.42 (t, *J* = 7.2 Hz, 2H), 7.36 – 7.26 (m, 4H), 5.66 (dd, *J* = 8.4, 6.8 Hz, 1H), 5.22 – 5.15 (m, 1H). ^{13}C NMR (151 MHz, DMSO) δ 134.63, 134.19, 133.99, 133.45, 132.74, 132.28, 132.20, 131.84, 131.78, 130.97, 130.76, 130.28, 130.14, 129.68, 129.60, 129.24, 129.16, 128.81, 128.73, 113.20, 113.13, 112.01, 111.91, 42.11, 41.83, 25.05. ^{31}P NMR (243 MHz, DMSO) δ 29.01.

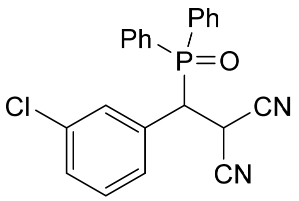

5c *(2-((3-chlorophenyl) (diphenylphosphoryl)methyl) malononitrile)*

White solid, yield: 165mg, 85%; ^{1}H NMR (400 MHz, Chloroform-*d*) δ 8.02 (dd, *J* = 11.6, 7.6 Hz, 2H), 7.75 – 7.63 (m, 3H), 7.59 – 7.44 (m, 3H), 7.36 (d, *J* = 7.2 Hz, 5H), 7.27 (d, *J* = 8.0 Hz, 1H), 4.74 (s, 1H), 4.03 (t, *J* = 7.6 Hz, 1H). ^{13}C NMR (151 MHz, $CDCl_3$) δ 135.15, 133.43, 131.32, 131.30, 131.20, 131.18, 130.53, 129.87, 129.65, 129.48, 129.41, 128.65, 128.57, 127.72, 110.94, 110.93, 47.07, 46.67, 24.62. ^{31}P NMR (243 MHz, $CDCl_3$) δ 28.36.

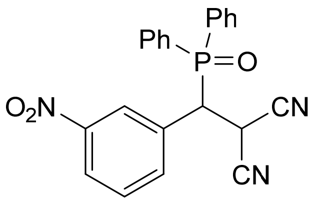

5d *(2-((diphenylphosphoryl)(3-nitrophenyl) methyl) malononitrile)*

White solid, yield: 164mg, 82%; ^{1}H NMR (400 MHz, Chloroform-*d*) δ 8.19 (d, *J* = 8.8 Hz, 2H), 8.09 – 8.02 (m, 2H), 7.96 (d, *J* = 7.6 Hz, 1H), 7.71 (ddd, *J* = 18.8, 7.6, 2.4 Hz, 3H), 7.57 (ddd, *J* = 11.2, 8.0, 2.0 Hz, 3H), 7.44 (d, *J* = 7.2 Hz, 1H), 7.35 (td, *J* = 7.6, 3.2 Hz, 2H), 4.75 (s, 1H), 4.20 (t, *J* = 7.2 Hz, 1H). ^{13}C NMR (151 MHz, $CDCl_3$) δ 135.18, 133.71, 133.09, 132.91, 131.33, 131.27, 130.95, 130.89, 130.47, 129.67, 129.59, 128.91, 128.83, 125.11, 124.25, 110.68, 46.98, 46.57, 29.71, 24.47. ^{31}P NMR (243 MHz, $CDCl_3$) δ 28.24.

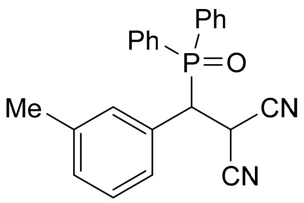

5e *(2-((diphenylphosphoryl)(m-tolyl) methyl) malononitrile)*

White solid, yield: 157mg, 85%; ^{1}H NMR (400 MHz, Chloroform-*d*) δ 8.03 – 7.97 (m, 2H), 7.66 (dtd, *J* = 14.8, 7.2, 2.4 Hz, 3H), 7.55 – 7.48 (m, 2H), 7.43 (dd, *J* = 7.6, 1.6 Hz, 1H), 7.33 (dd, *J* = 7.6, 3.6 Hz, 2H), 7.22 – 7.11 (m, 4H), 4.83 (s, 1H), 4.03 (t, *J* = 8.0 Hz, 1H), 2.28 (s, 3H). ^{13}C NMR (151 MHz, $CDCl_3$) δ 133.17, 132.69, 132.37, 131.36, 130.58, 130.30, 130.13, 129.30, 129.22, 129.12, 128.88, 128.35, 128.27, 126.77, 111.31, 111.30, 47.42, 47.00, 24.70, 21.30. ^{31}P NMR (243 MHz, $CDCl_3$) δ 28.64.

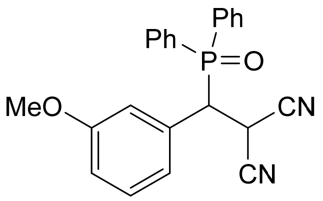

5f *(2-((diphenylphosphoryl)(3-methoxyphenyl) methyl) malononitrile)*

White solid, yield: 156mg, 81%; ^{1}H NMR (400 MHz, Chloroform-*d*) δ 8.04 – 7.95 (m, 2H), 7.71 – 7.53 (m, 5H), 7.45 (d, *J* = 1.6 Hz, 1H), 7.33 (d, *J* = 3.2 Hz, 2H), 7.22 (d, *J* = 8.0 Hz, 1H), 7.01 – 6.92 (m, 2H), 6.86 (dd, *J* = 8.4, 2.4 Hz, 1H), 4.77 (s, 1H), 4.05 (t, *J* = 8.0 Hz, 1H), 3.73 (s, 3H). ^{13}C NMR (151 MHz, $CDCl_3$) δ 133.20, 132.46, 132.03, 131.36, 131.36, 130.31, 129.65, 129.35, 129.27, 128.85, 128.45, 128.46, 128.38, 122.08, 115.48, 114.83, 111.28, 55.29, 47.43, 47.01, 24.78. ^{31}P NMR (243 MHz, $CDCl_3$) δ 30.84.

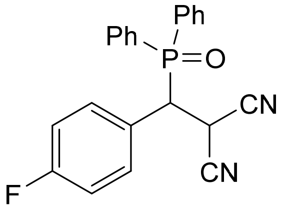

5g *2-((diphenylphosphoryl)(4-fluorophenyl) methyl) malononitrile*

White solid, yield: 145mg, 78%; ^{1}H NMR (400 MHz, DMSO-d_6) δ 8.17 – 8.09 (m, 2H), 7.69 – 7.62 (m, 5H), 7.53 (s, 4H), 7.44 – 7.32 (m, 3H), 5.46 (t, *J* = 7.3 Hz, 1H), 5.17 (t, *J* = 7.1 Hz, 1H). ^{13}C NMR (151 MHz, DMSO) δ 133.11, 132.60, 132.46, 132.39, 132.32, 131.88, 131.62, 131.56, 131.37, 131.30, 130.82, 130.76, 130.16, 130.10, 129.49, 129.42, 128.87, 128.79, 116.00, 115.85, 115.09, 114.95, 113.17, 112.93, 79.57, 79.35, 79.14, 42.23, 41.80, 25.47. ^{31}P NMR (243 MHz, DMSO) δ 28.58. ^{19}F NMR (565 MHz, DMSO) δ -112.90.

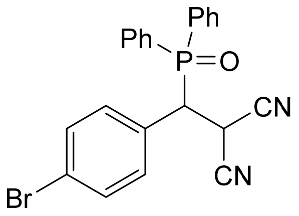

5h *2-((4-bromophenyl) (diphenylphosphoryl)methyl) malononitrile*

White solid, yield: 171mg, 79%; ^{1}H NMR (400 MHz, Chloroform-*d*) δ 8.03 – 7.97 (m, 2H), 7.83 – 7.50 (m, 7H), 7.44 (dtd, *J* = 8.4, 3.9, 2.0 Hz, 3H), 7.35 (td, *J* = 7.6, 3.2 Hz, 1H), 7.02 (t, *J* = 8.6 Hz, 1H), 4.69 (t, *J* = 7.2 Hz, 1H), 4.05 (t, *J* = 7.6 Hz, 1H). ^{13}C NMR (151 MHz, $CDCl_3$) δ 137.03, 136.79, 133.15, 132.51, 131.97, 131.28, 130.69, 130.43, 130.25, 129.81, 129.27, 128.80, 128.45, 111.89, 111.37, 78.00, 77.95, 42.23, 41.80, 25.47. ^{31}P NMR (243 MHz, $CDCl_3$) δ 29.69.

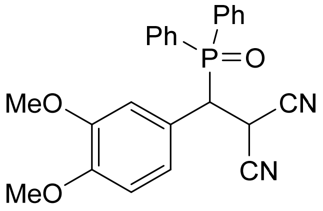

5i *(2-((3,4-dimethoxyphenyl) (diphenylphosphoryl)methyl) malononitrile)*

White solid, yield: 174mg, 84%; ^{1}H NMR (400 MHz, Chloroform-*d*) δ 8.02 – 7.92 (m, 2H), 7.72 – 7.53 (m, 5H), 7.47 (t, *J* = 7.6 Hz, 1H), 7.36 (td, *J* = 7.6, 2.8 Hz, 2H), 6.96 (d, *J* = 5.6 Hz, 2H), 6.82 (d, *J* = 8.4 Hz, 1H), 4.73 (s, 1H), 4.04 – 3.96 (m, 1H), 3.88 (s, 3H), 3.77 (s, 3H). ^{13}C NMR (151 MHz, $CDCl_3$) δ 139.74, 139.53, 133.21, 132.49, 131.88, 131.31, 131.25, 131.09, 131.04, 130.74, 130.39, 129.73, 129.65, 129.31, 128.94, 128.86, 78.00, 77.95, 47.42, 47.01, 24.70. ^{31}P NMR (243 MHz, $CDCl_3$) δ 28.47.

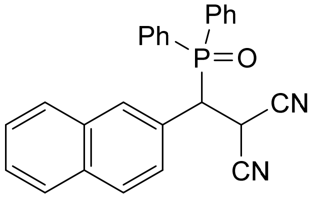

5j *(2-((diphenylphosphoryl)(naphthalen-2-yl) methyl) malononitrile)*

White solid, yield: 142mg, 70%; ^{1}H NMR (400 MHz, Chloroform-*d*) δ 8.03 (dd, *J* = 11.6, 7.6 Hz, 2H), 7.91 (s, 1H), 7.81 (t, *J* = 9.2 Hz, 4H), 7.73 – 7.61 (m, 3H), 7.58 – 7.46 (m, 5H), 7.25 (td, *J* = 7.6, 3.2 Hz, 2H), 4.85 (t, *J* = 7.6 Hz, 1H), 4.23 (t, *J* = 8.0 Hz, 1H). ^{13}C NMR (151 MHz, $CDCl_3$) δ 133.23, 133.11, 132.45, 132.02, 131.35, 131.29, 130.38, 129.72, 129.49, 129.38, 129.31, 129.26, 128.79, 128.46, 128.38, 128.18, 127.72, 127.07, 126.80, 126.48, 111.36, 111.30, 111.21, 47.54, 47.12, 24.97. ^{31}P NMR (243 MHz, $CDCl_3$) δ 28.51.

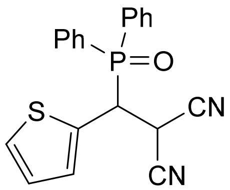

5k *(2-((diphenylphosphoryl)(thiophen-2-yl) methyl) malononitrile)*

White solid, yield: 130mg, 72%; ^{1}H NMR (400 MHz, Chloroform-*d*) δ 7.96 (q, *J* = 7.6, 6.4 Hz, 2H), 7.86 – 7.74 (m, 2H), 7.70 (d, *J* = 7.2 Hz, 1H), 7.64 (d, *J* = 7.6 Hz, 4H), 7.52 (s, 1H), 7.41 (d, *J* = 7.2 Hz, 2H), 7.25 (d, *J* = 3.2 Hz, 1H), 4.75 (s, 1H), 4.40 (s, 1H). ^{13}C NMR (151 MHz, $CDCl_3$) δ 133.37, 132.78, 132.35, 131.89, 131.64, 131.58, 131.41, 131.34, 130.80, 130.68, 129.81, 129.42, 129.34, 129.16, 128.87, 128.55, 128.47, 128.36, 128.16, 127.85, 127.45, 126.82, 111.25, 111.19, 110.96, 110.92, 42.97, 42.54, 25.62. ^{31}P NMR (243 MHz, $CDCl_3$) δ 28.36.

Spectra of Products

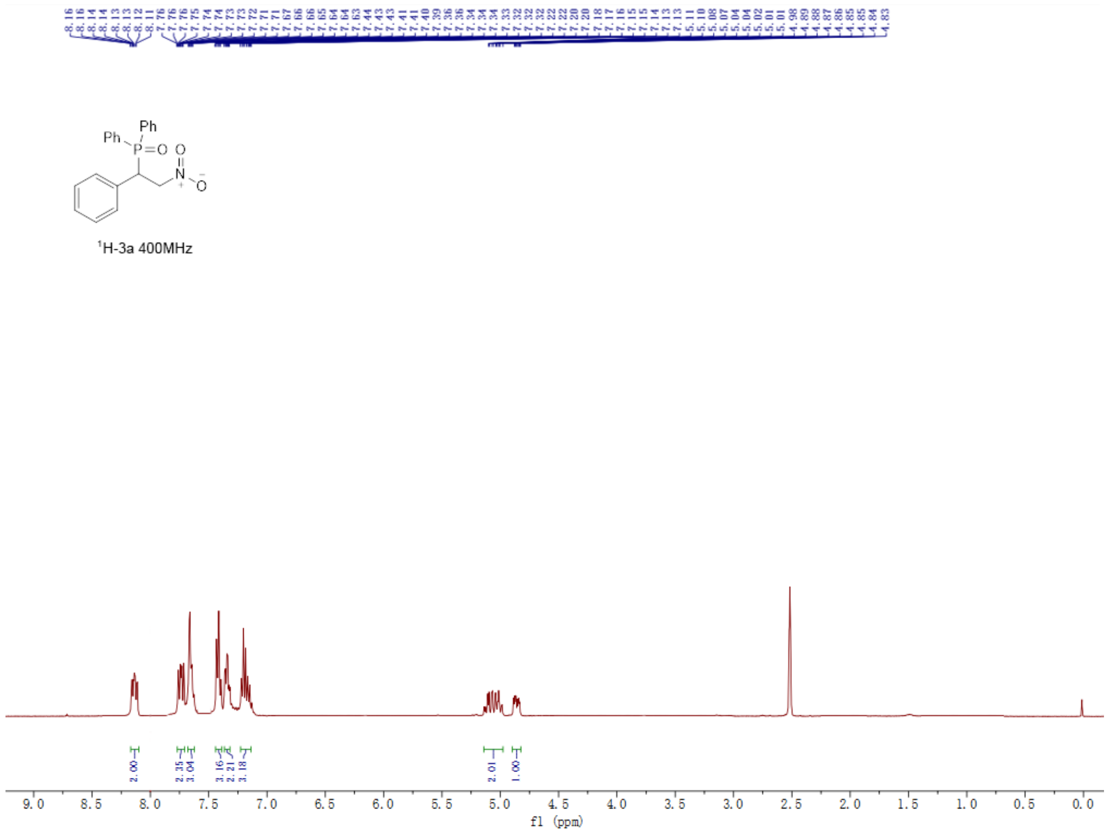

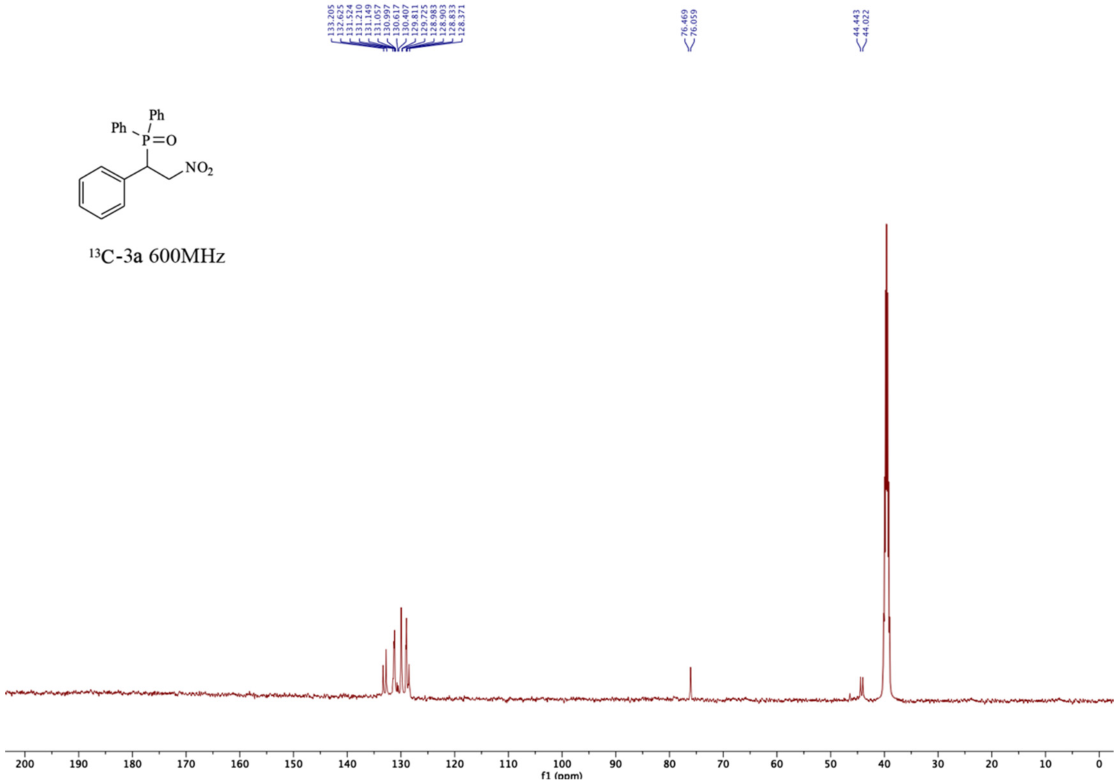

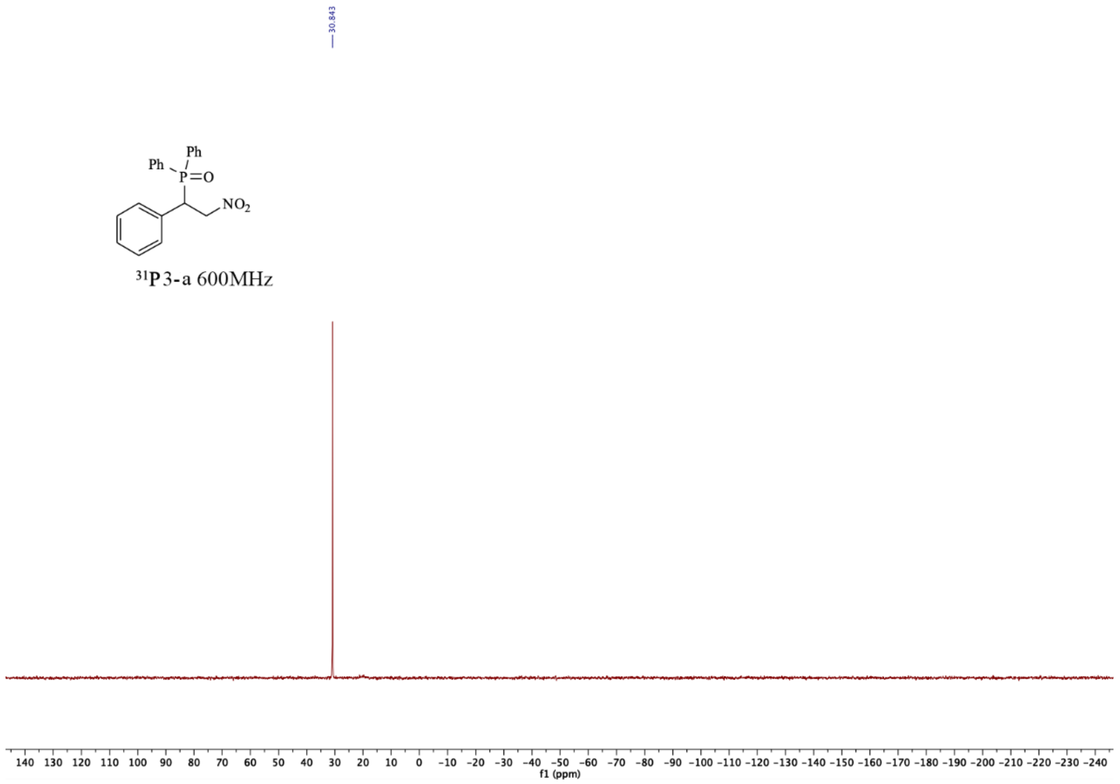

^{31}P 3-a 600MHz

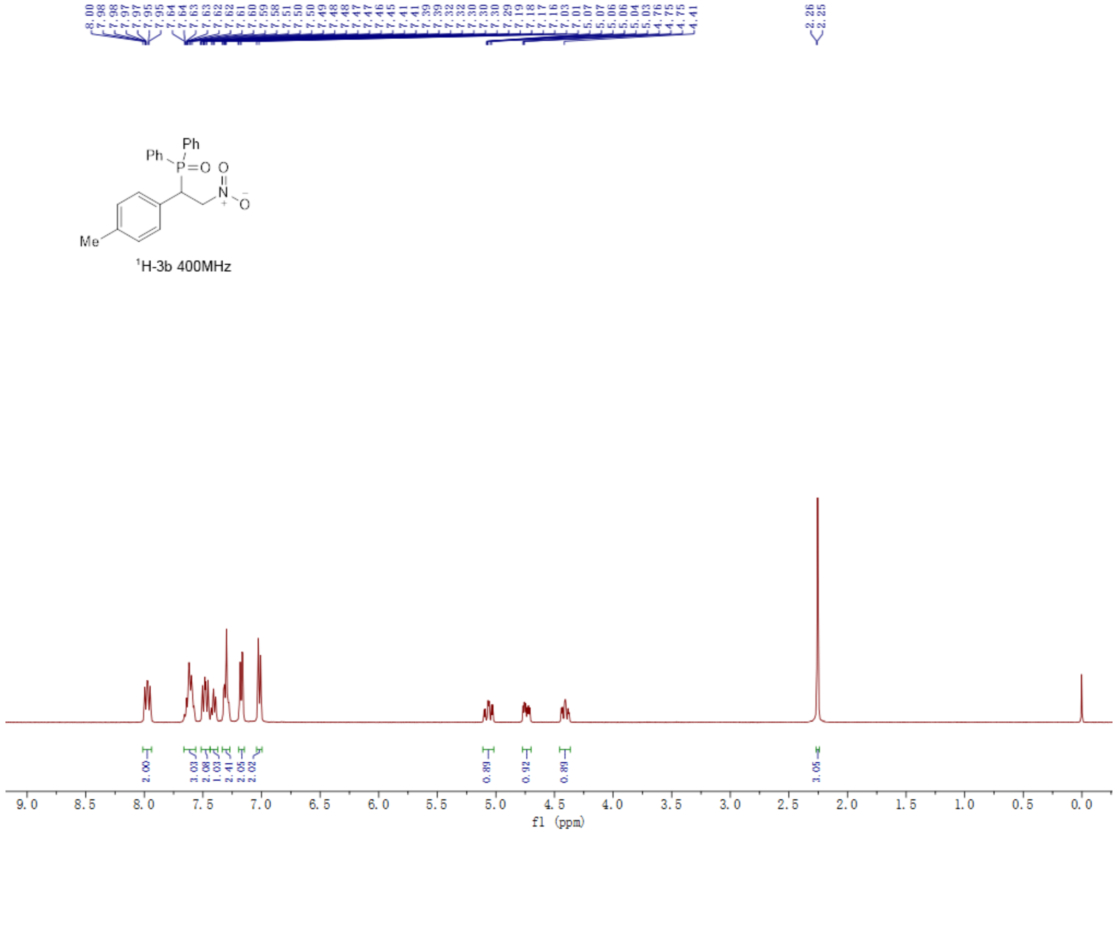

^{1}H-3b 400MHz

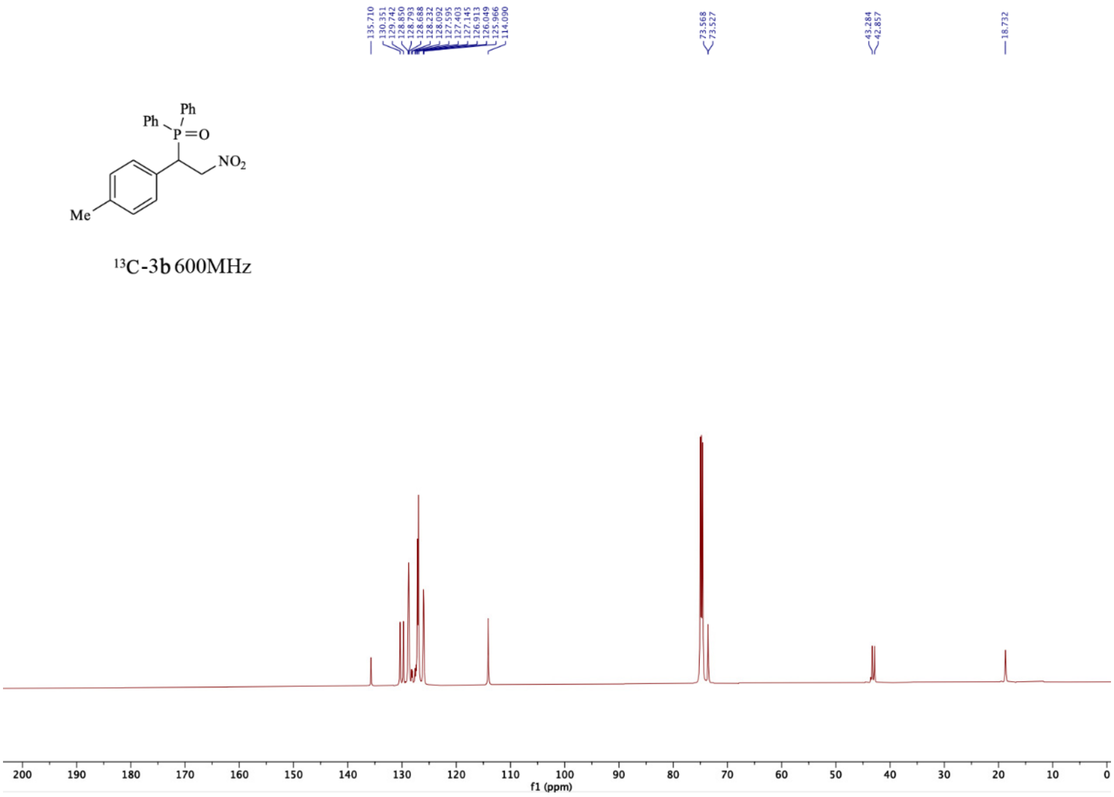

^{13}C-3b 600MHz

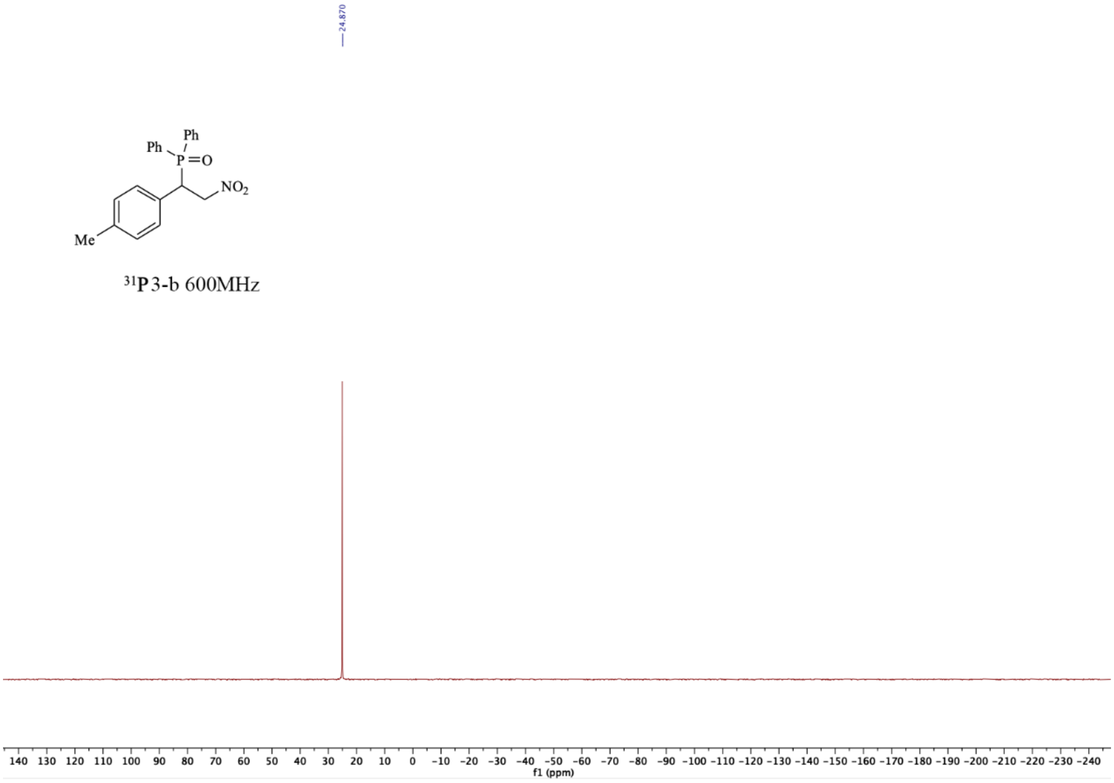

^{31}P 3-b 600MHz

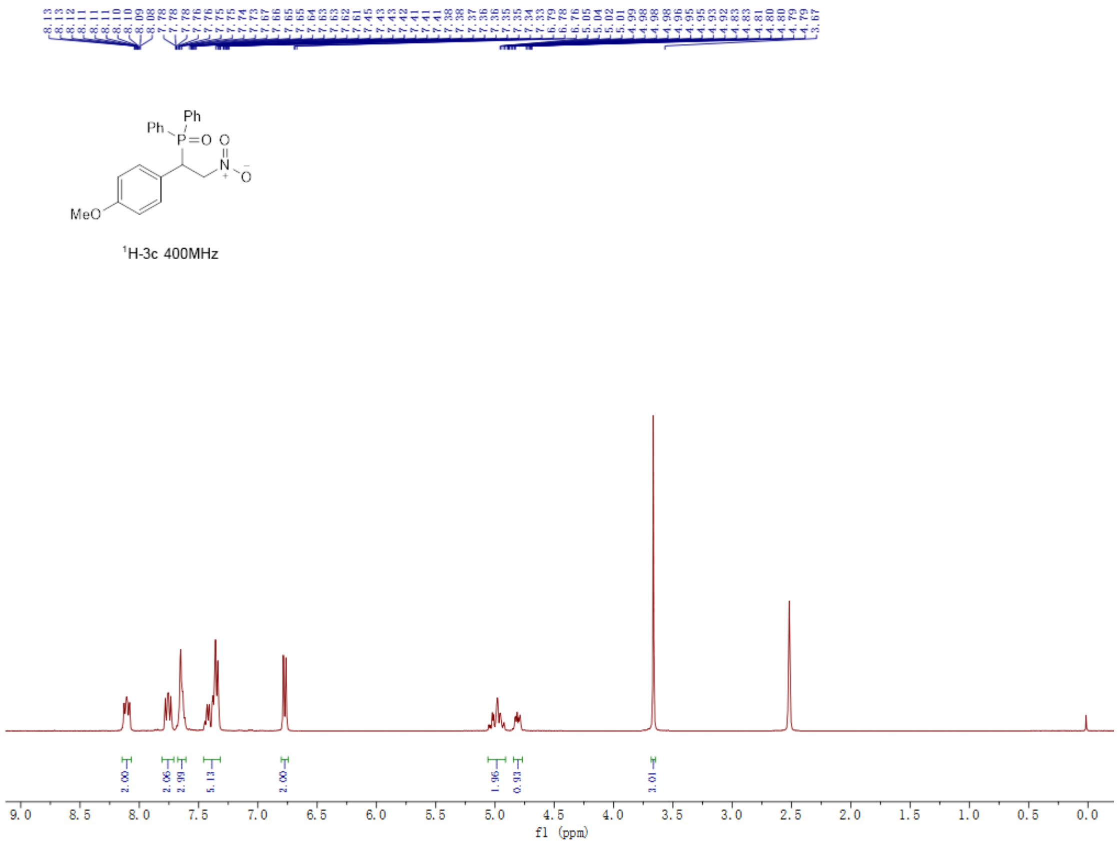

¹H-3c 400MHz
f1 (ppm)

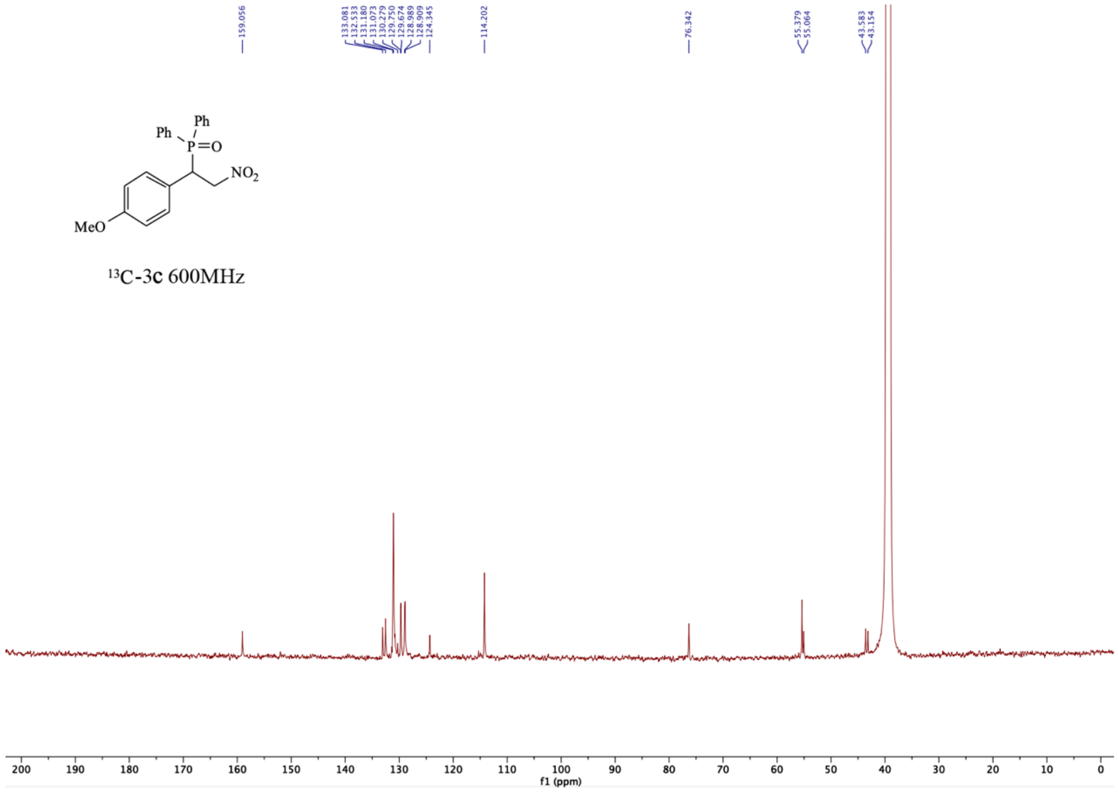

¹³C-3c 600MHz
f1 (ppm)

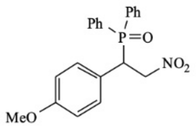

^{31}P 3-c 600MHz

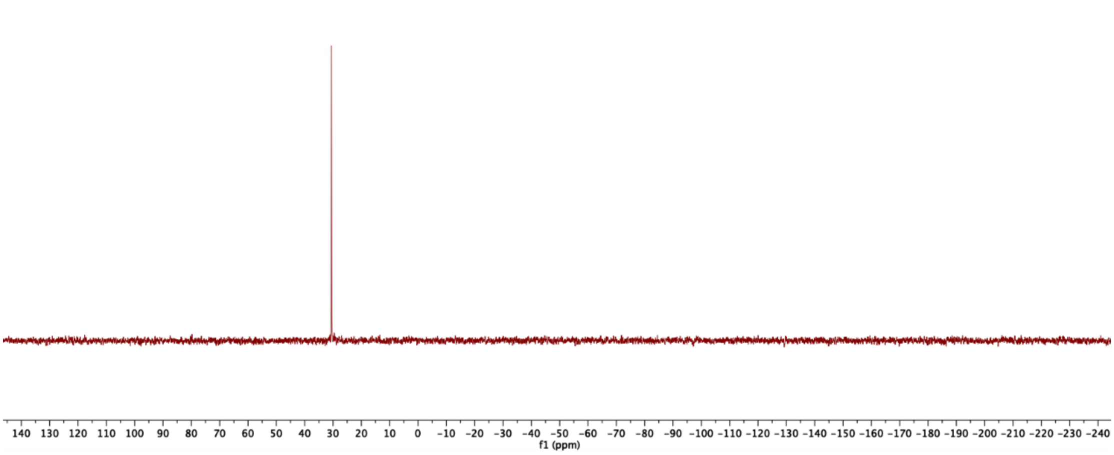

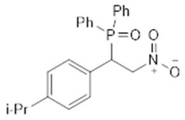

^{1}H-3d 400MHz

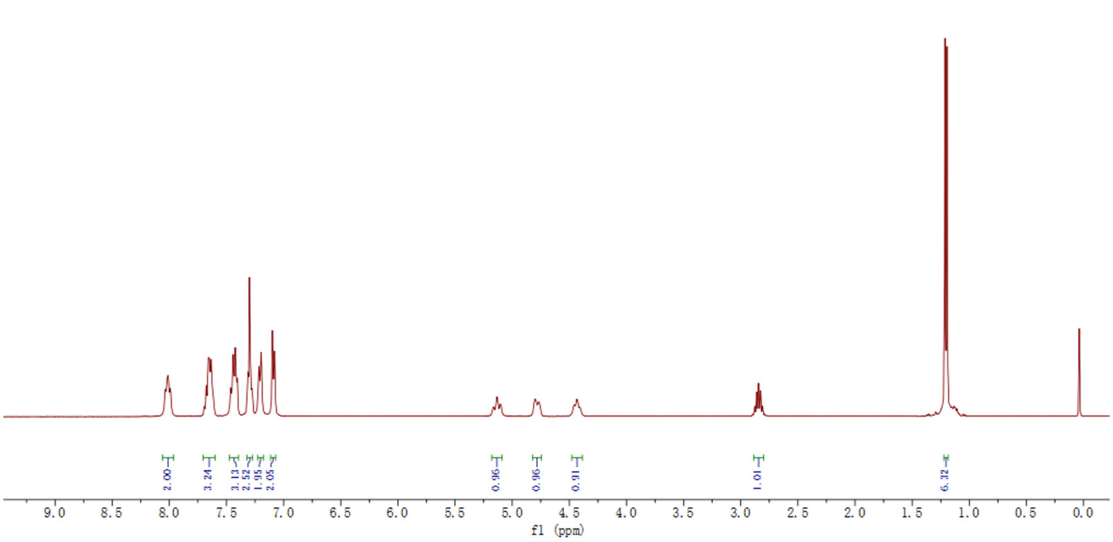

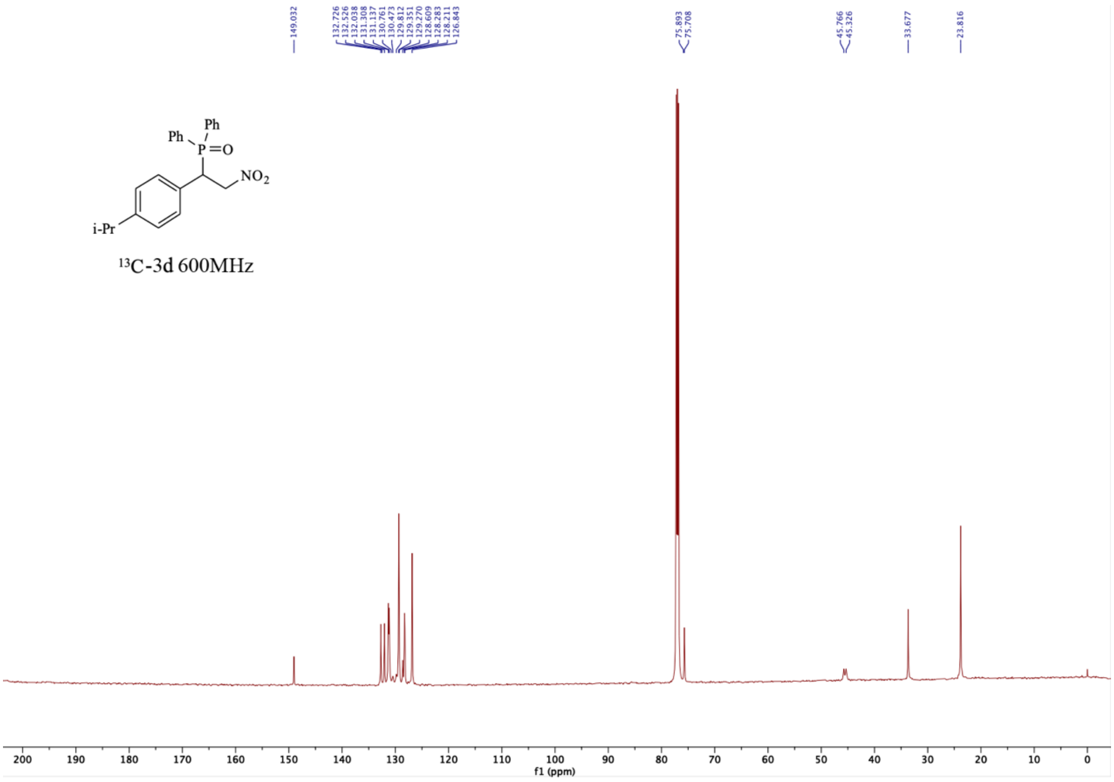

Ph
Ph
P=O
NO2
i-Pr
13C-3d 600MHz
149.032
132.726
132.526
132.038
131.308
131.137
130.761
130.473
129.812
129.351
129.270
128.609
128.283
128.211
126.843
75.893
75.708
45.766
45.326
33.677
23.816
f1 (ppm)

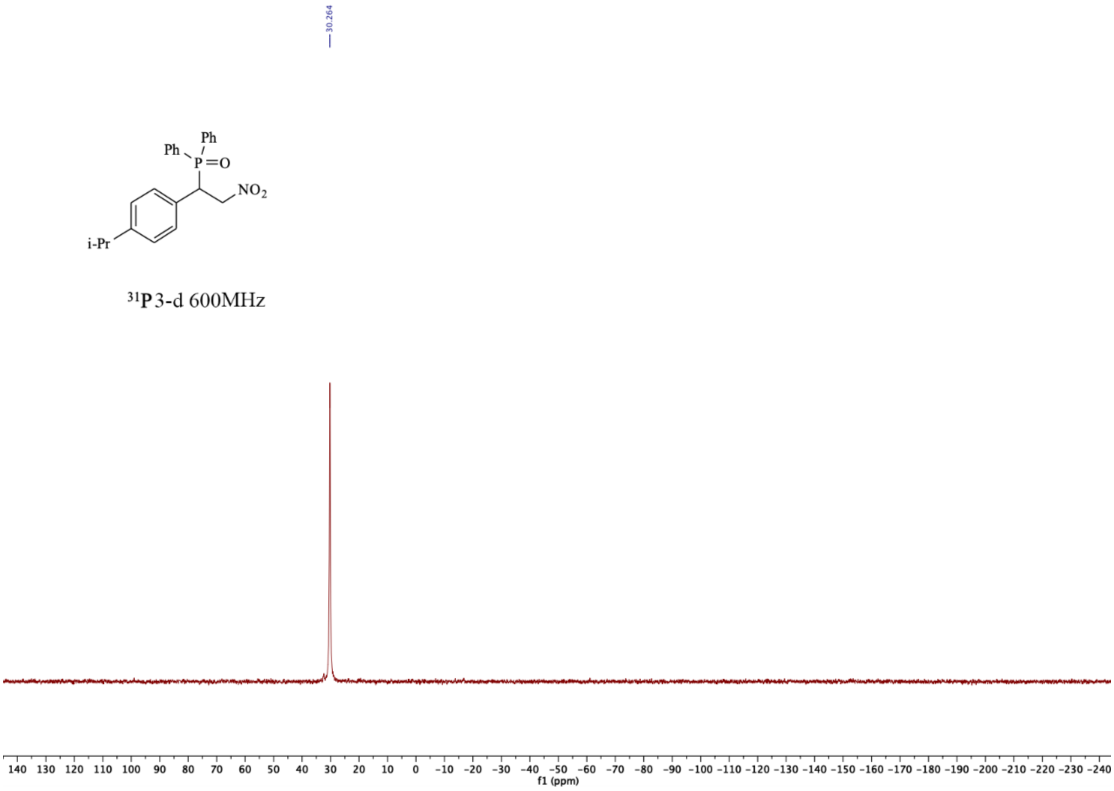

30.264
Ph
Ph
P=O
NO2
i-Pr
31P 3-d 600MHz
f1 (ppm)

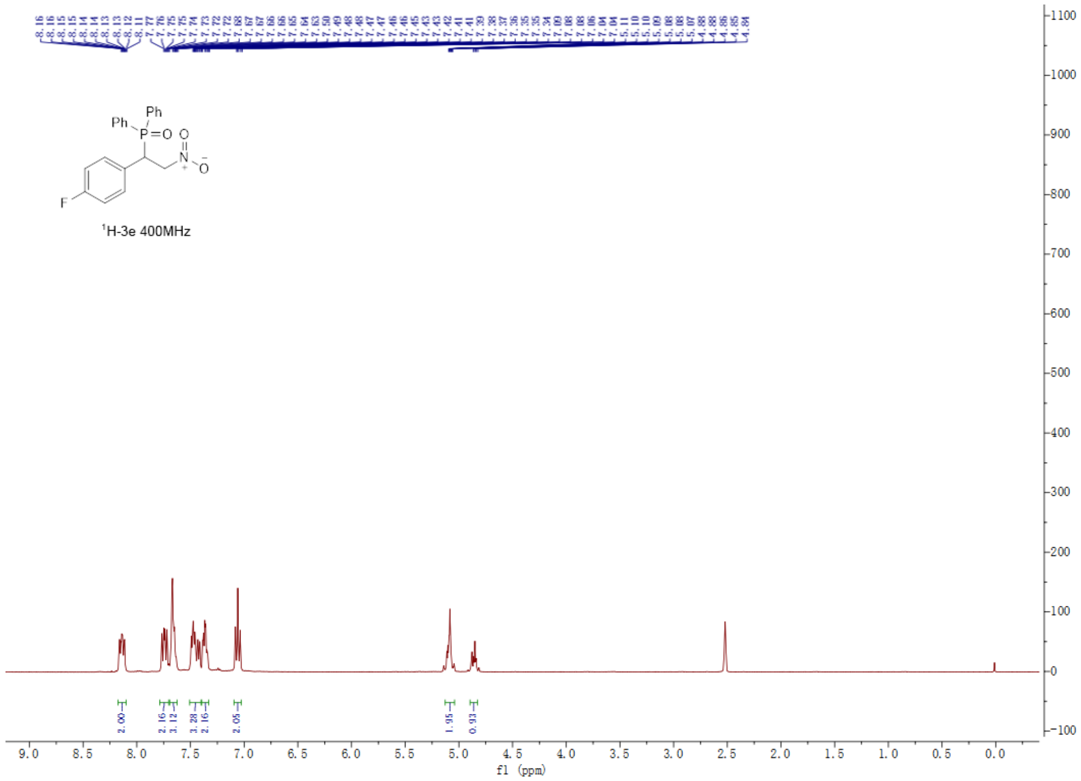
Ph
Ph
P=O
F
^{1}H-3e 400MHz
f1 (ppm)

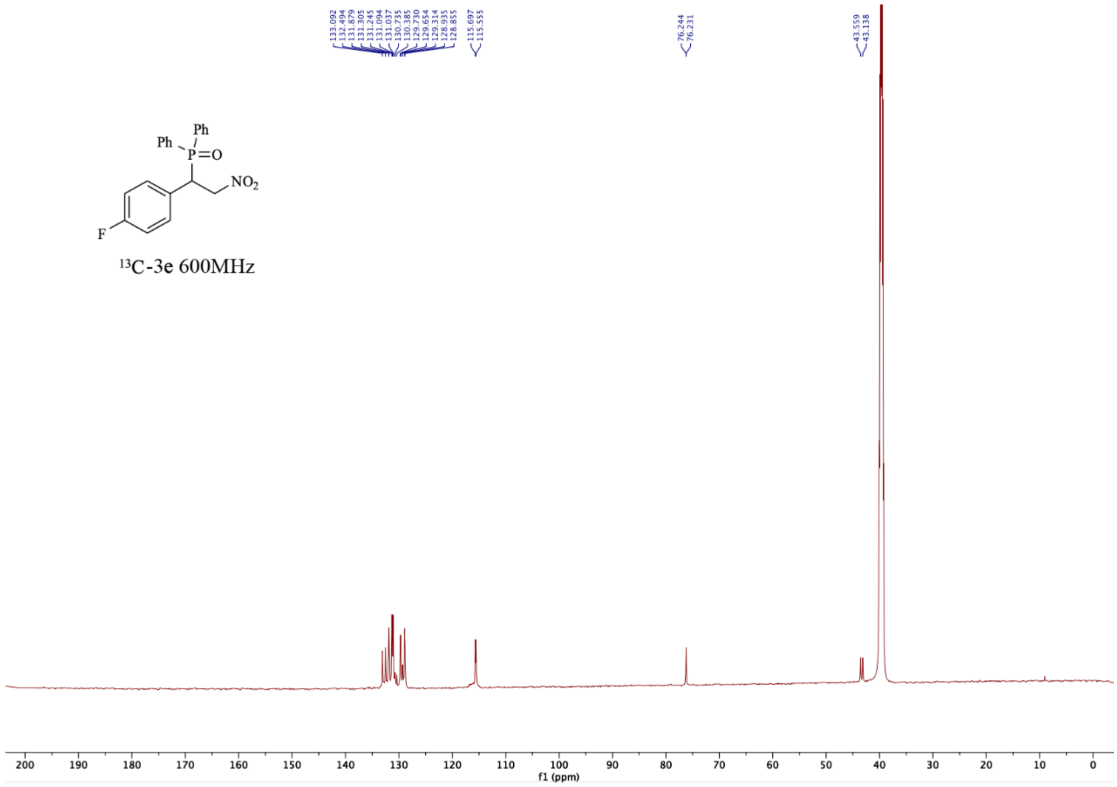
Ph
Ph
P=O
NO2
F
13C-3e 600MHz
f1 (ppm)

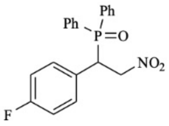

^{31}P 3-e 600MHz

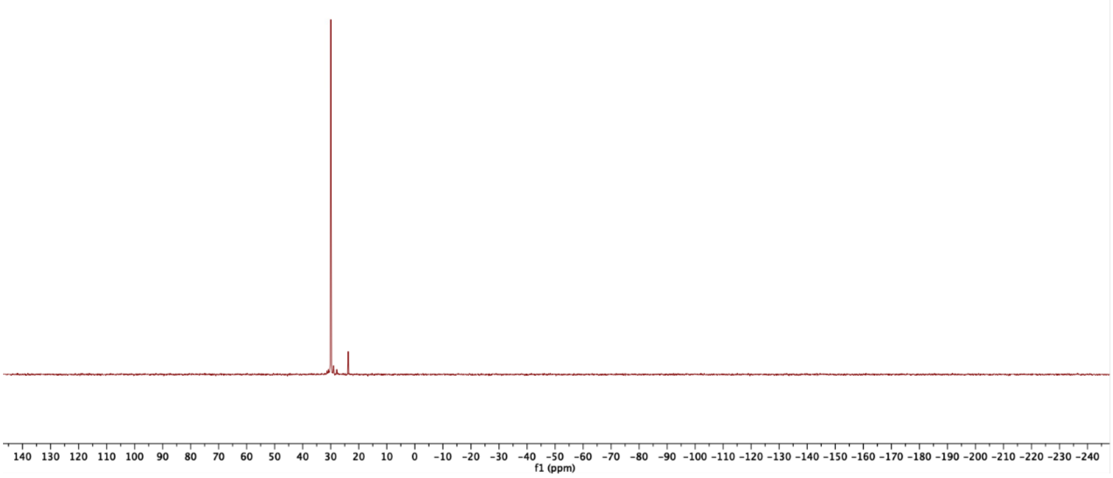

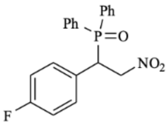

^{19}F 3-e 600 MHz

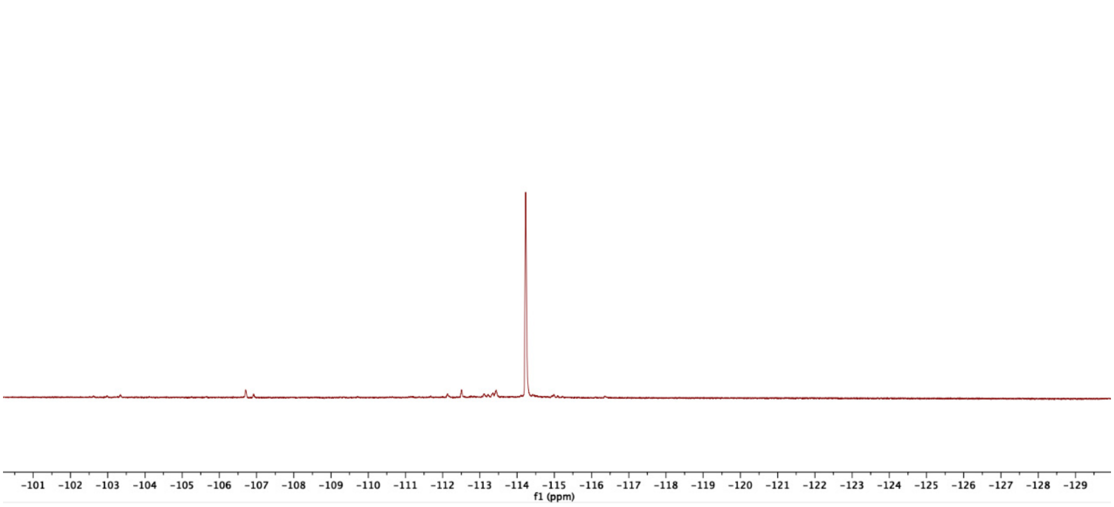

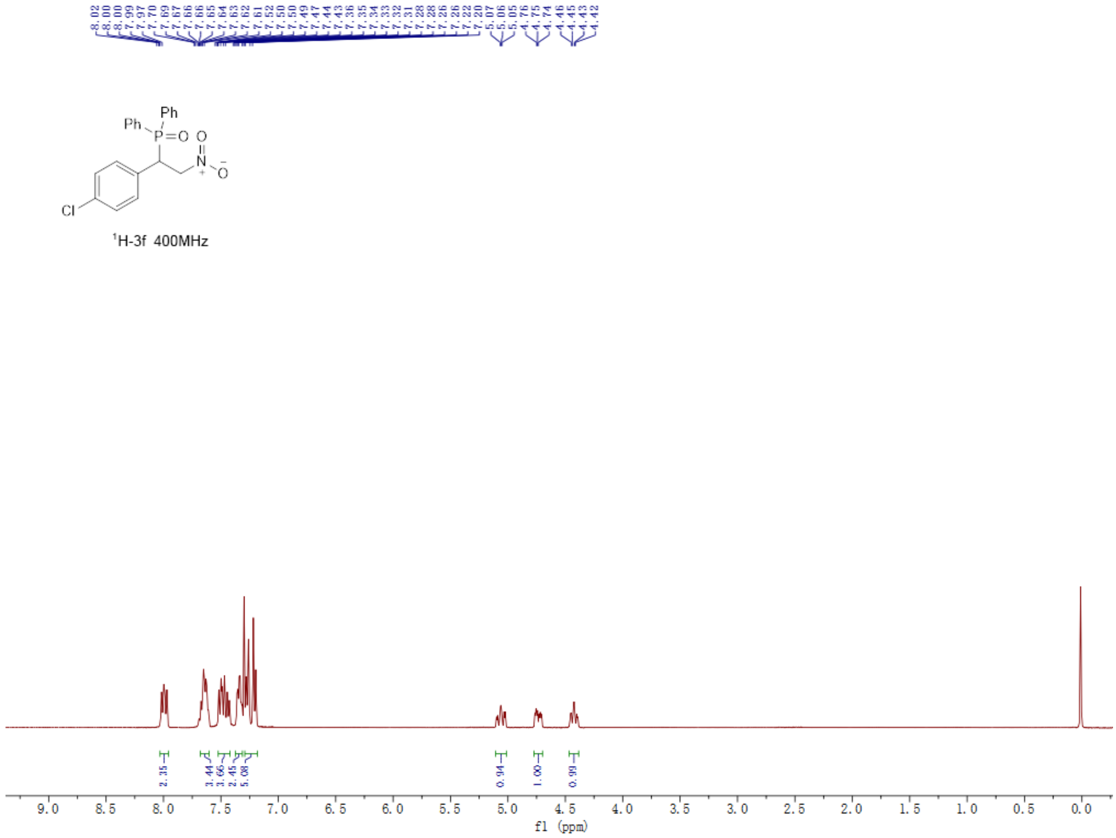
8.02 8.00 8.00 7.99 7.97 7.70 7.69 7.67 7.66 7.65 7.64 7.63 7.62 7.61 7.52 7.50 7.50 7.49 7.47 7.44 7.43 7.36 7.35 7.34 7.33 7.32 7.31 7.28 7.28 7.26 7.26 7.22 7.20 5.07 5.06 5.05 4.76 4.75 4.74 4.46 4.45 4.43 4.42
Ph
Ph
P=O
O
N
O
Cl
1H-3f 400MHz
2.35
3.44
3.66
2.45
5.08
0.94
1.00
0.99
9.0 8.5 8.0 7.5 7.0 6.5 6.0 5.5 5.0 4.5 4.0 3.5 3.0 2.5 2.0 1.5 1.0 0.5 0.0
f1 (ppm)

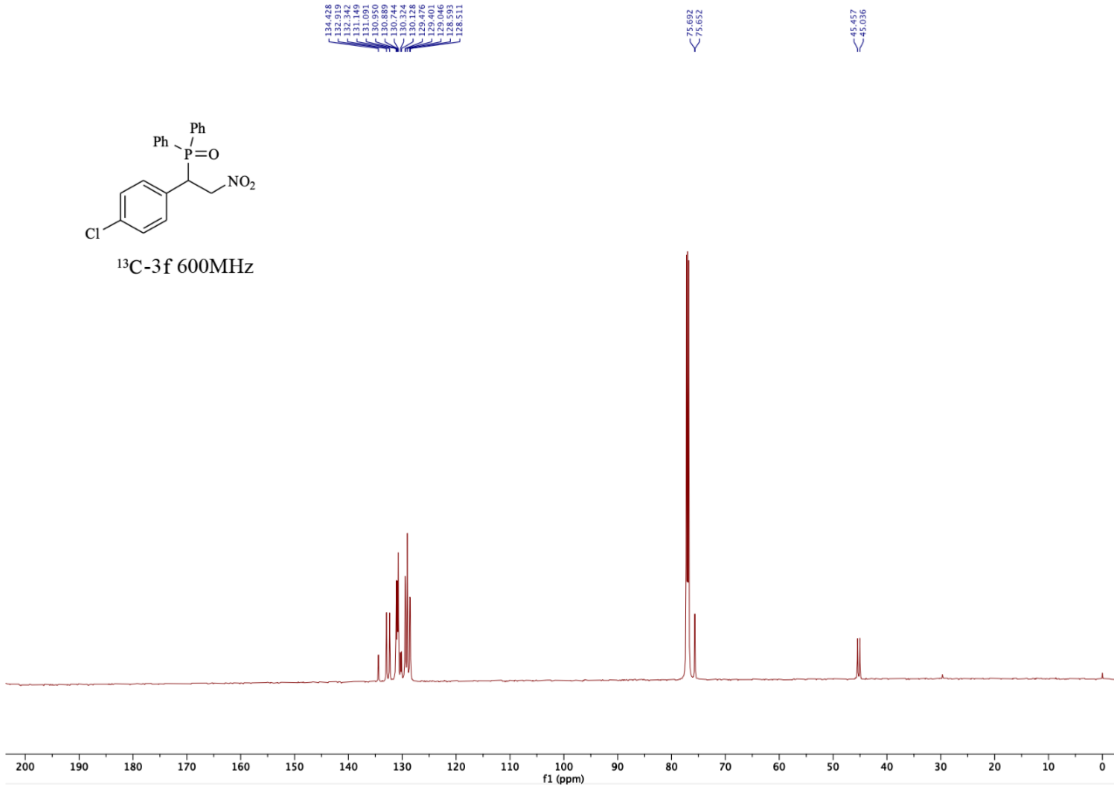
134.428 132.919 132.342 131.149 131.091 130.950 130.889 130.744 130.324 130.128 129.476 129.401 129.046 128.593 128.511
75.692 75.652
45.457 45.036
Ph
Ph
P=O
NO2
Cl
13C-3f 600MHz
200 190 180 170 160 150 140 130 120 110 100 90 80 70 60 50 40 30 20 10 0
f1 (ppm)

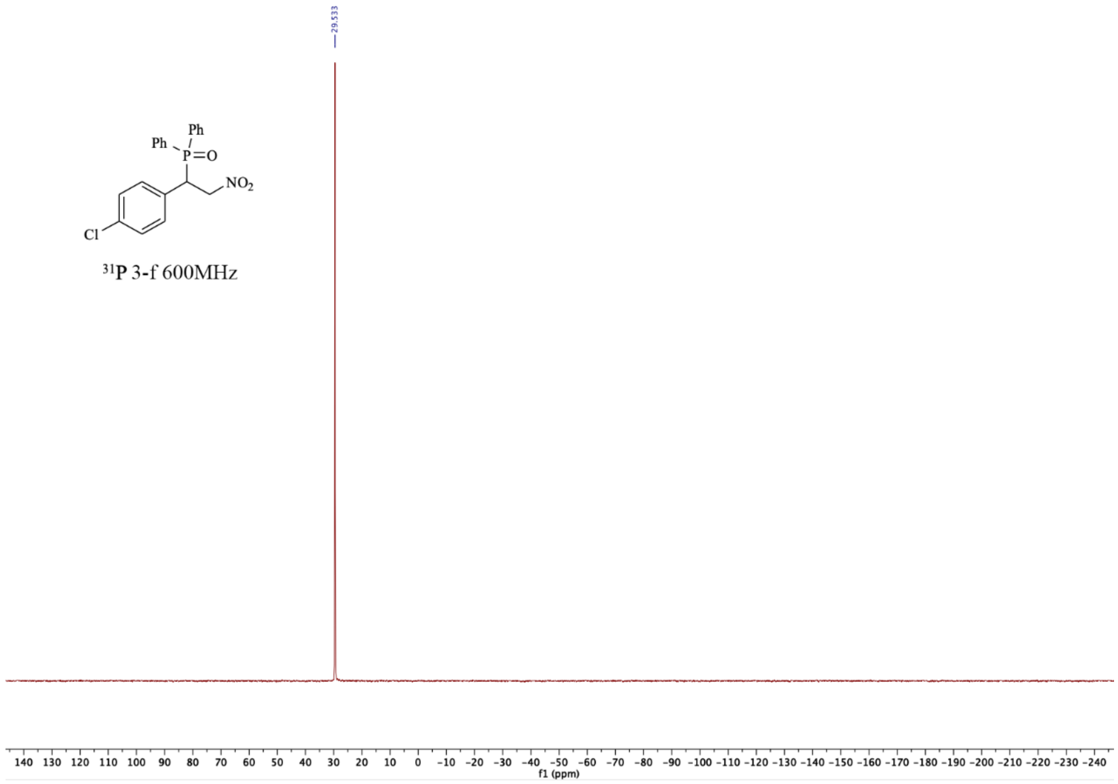

29.533
Ph
Ph
P=O
NO2
Cl
31P 3-f 600MHz
f1 (ppm)

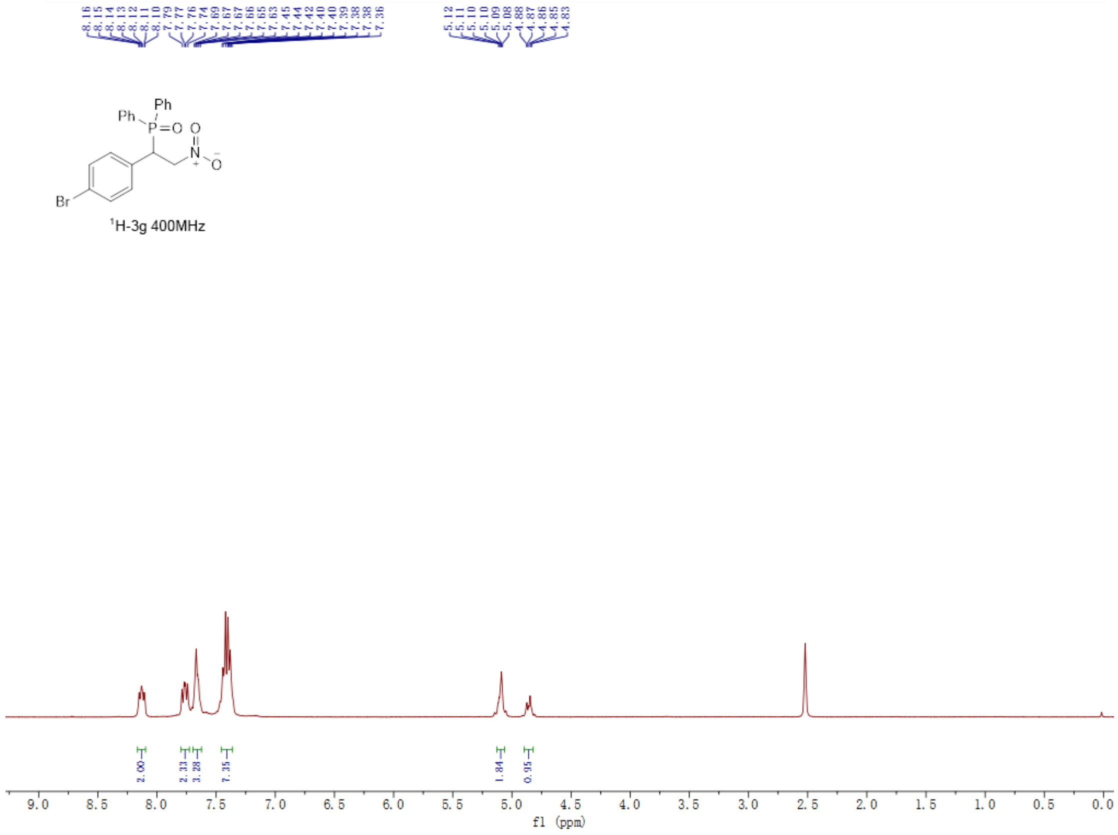

Ph
Ph
P=O
Br
1H-3g 400MHz
2.00
2.33
3.28
7.35
1.84
0.95
f1 (ppm)

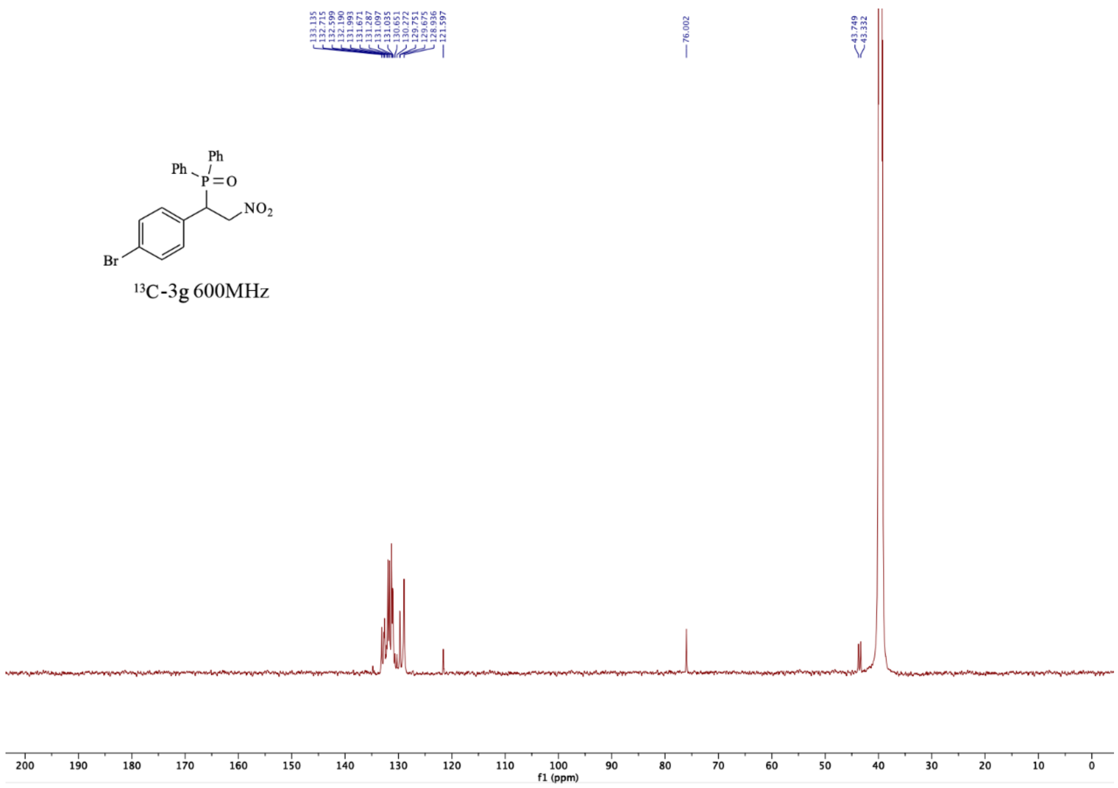

133.135
132.715
132.599
132.190
131.993
131.671
131.287
131.097
131.035
130.651
130.272
129.751
129.675
128.936
121.597
76.002
43.749
43.332
Ph
Ph
P=O
NO2
Br
13C-3g 600MHz
200 190 180 170 160 150 140 130 120 110 100 90 80 70 60 50 40 30 20 10 0
f1 (ppm)

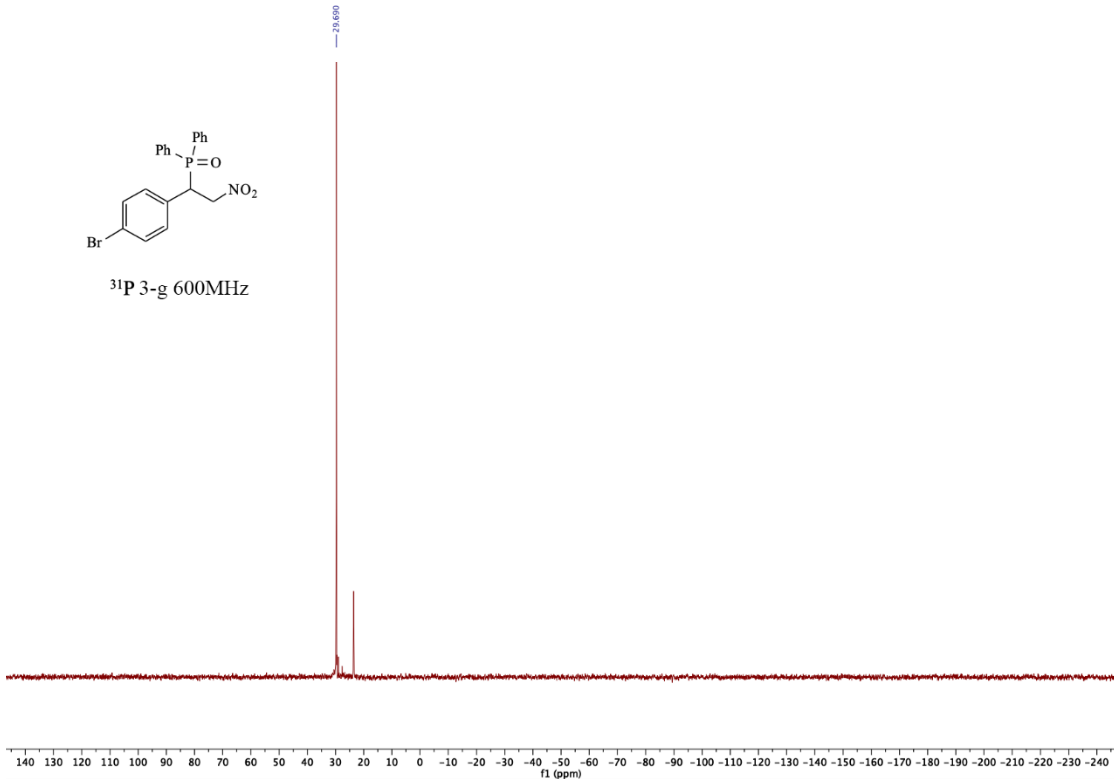

29.690
Ph
Ph
P=O
NO2
Br
31P 3-g 600MHz
140 130 120 110 100 90 80 70 60 50 40 30 20 10 0 -10 -20 -30 -40 -50 -60 -70 -80 -90 -100 -110 -120 -130 -140 -150 -160 -170 -180 -190 -200 -210 -220 -230 -240
f1 (ppm)

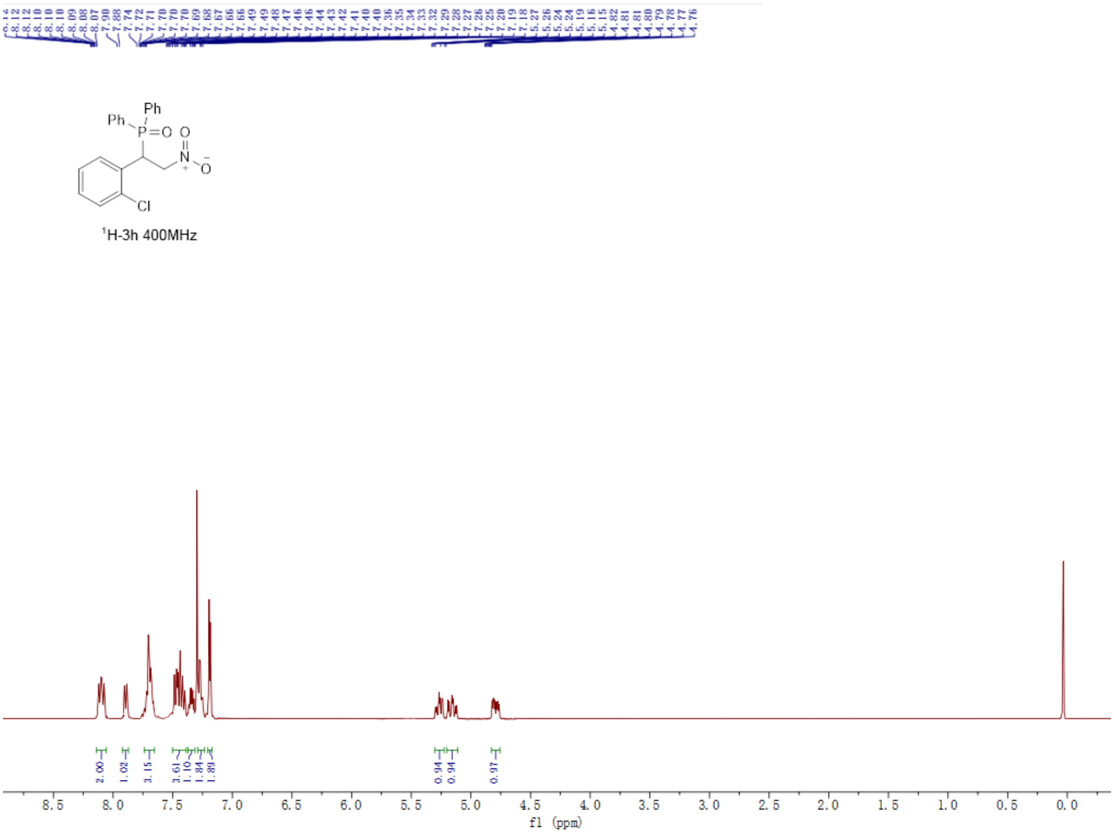
Ph
Ph
P=O
Cl
^{1}H-3h 400MHz
f1 (ppm)

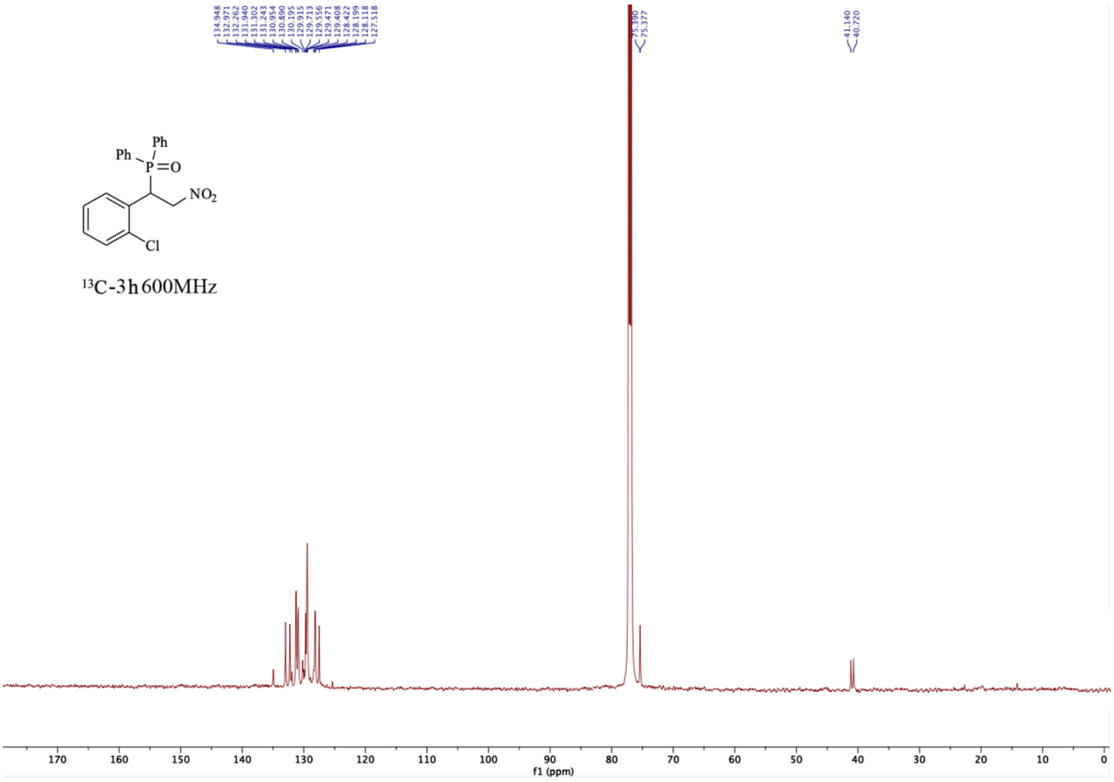
Ph
Ph
P=O
NO2
Cl
^{13}C-3h 600MHz
f1 (ppm)

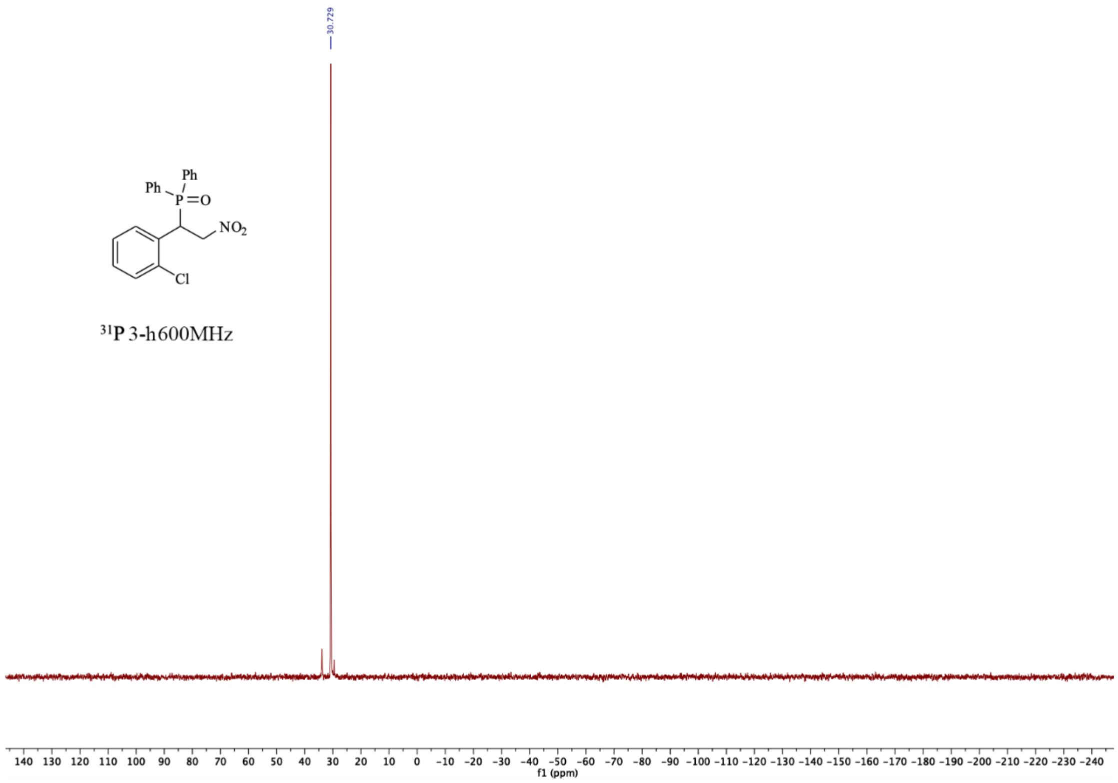
30.729
Ph
Ph
P=O
NO2
Cl
31P 3-h600MHz
f1 (ppm)

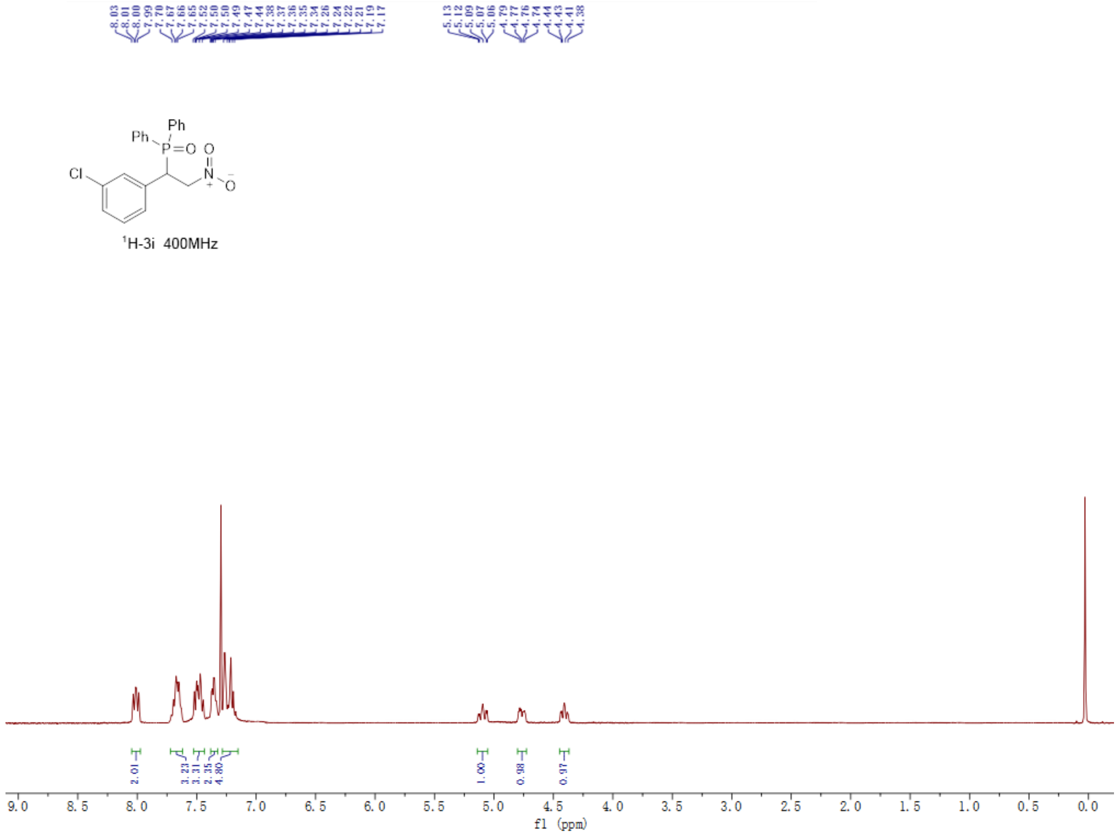
Ph
Ph
P=O
Cl
1H-3i 400MHz
f1 (ppm)

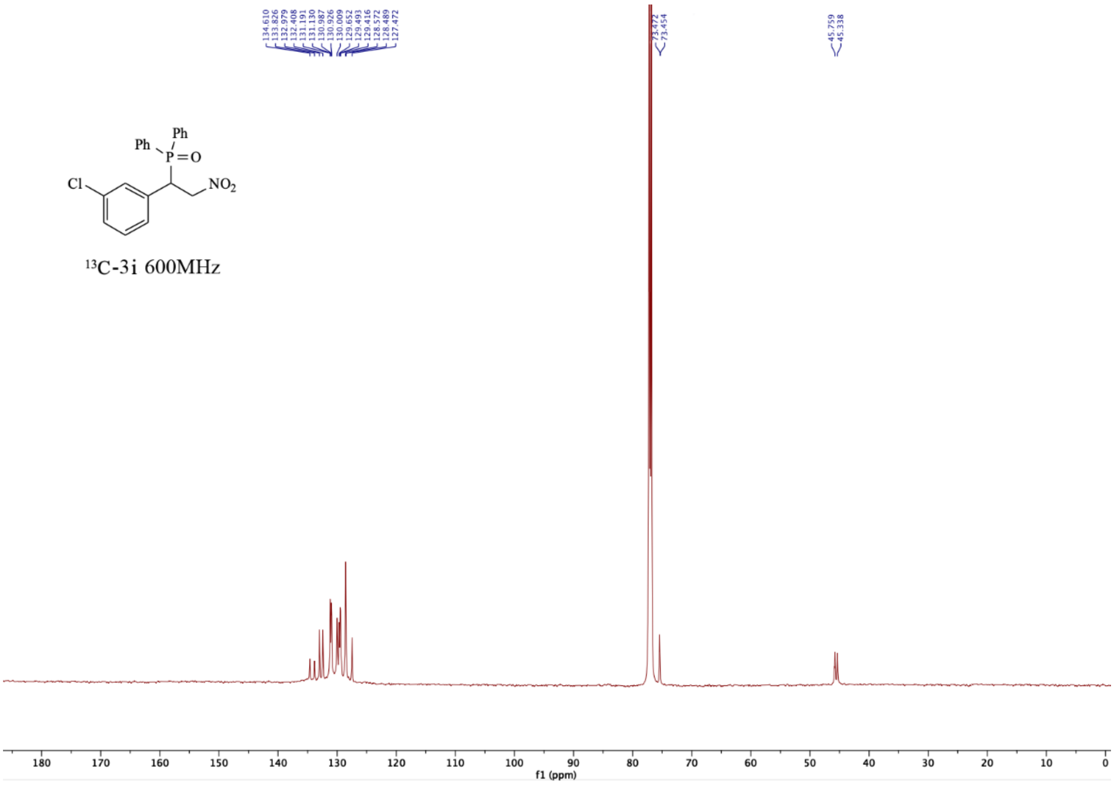

^{13}C-3i 600MHz

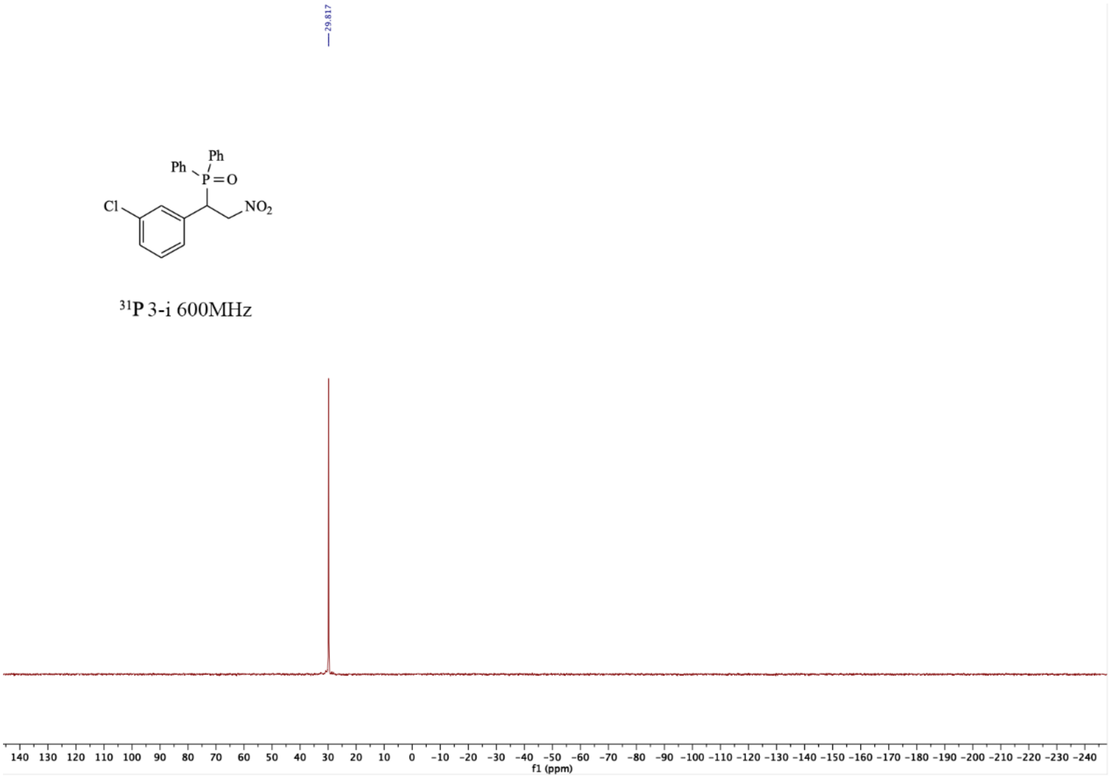

^{31}P 3-i 600MHz

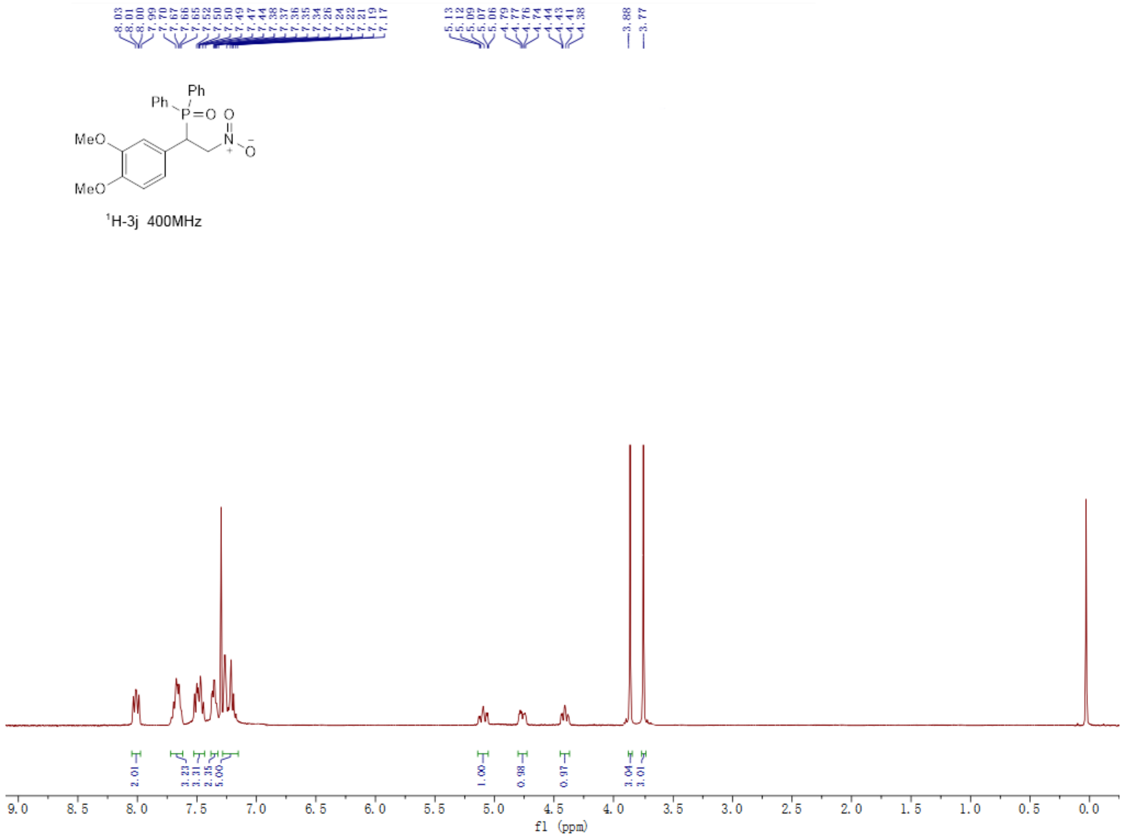

Ph
Ph
P=O
MeO
MeO
¹H-3j 400MHz
f1 (ppm)

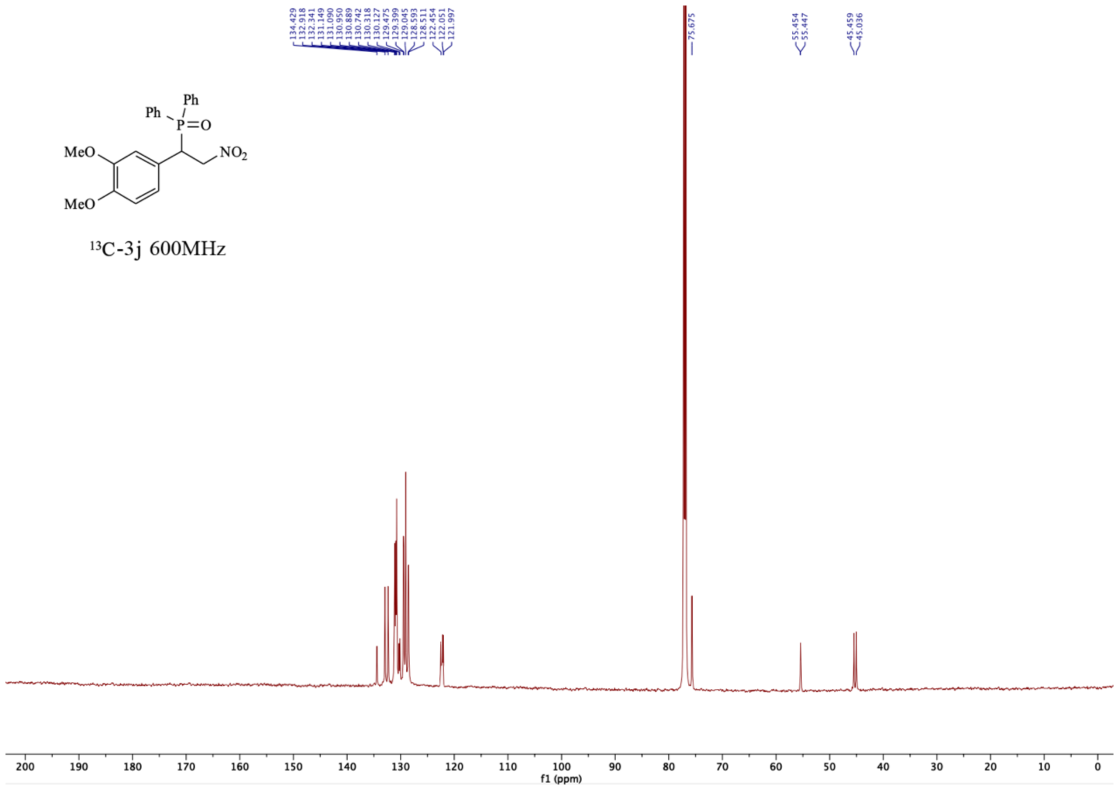

Ph
Ph
P=O
NO2
MeO
MeO
¹³C-3j 600MHz
f1 (ppm)

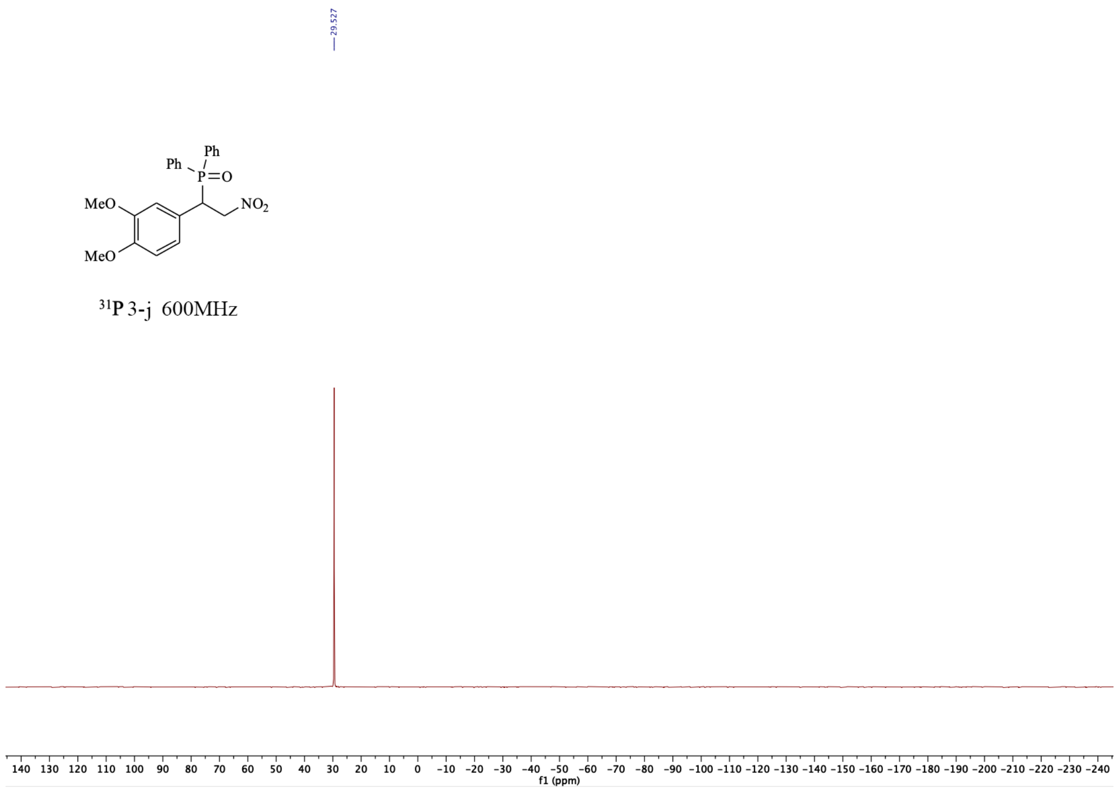
29.527
Ph
Ph
P=O
MeO
NO2
MeO
31P 3-j 600MHz
140 130 120 110 100 90 80 70 60 50 40 30 20 10 0 -10 -20 -30 -40 -50 -60 -70 -80 -90 -100 -110 -120 -130 -140 -150 -160 -170 -180 -190 -200 -210 -220 -230 -240
f1 (ppm)

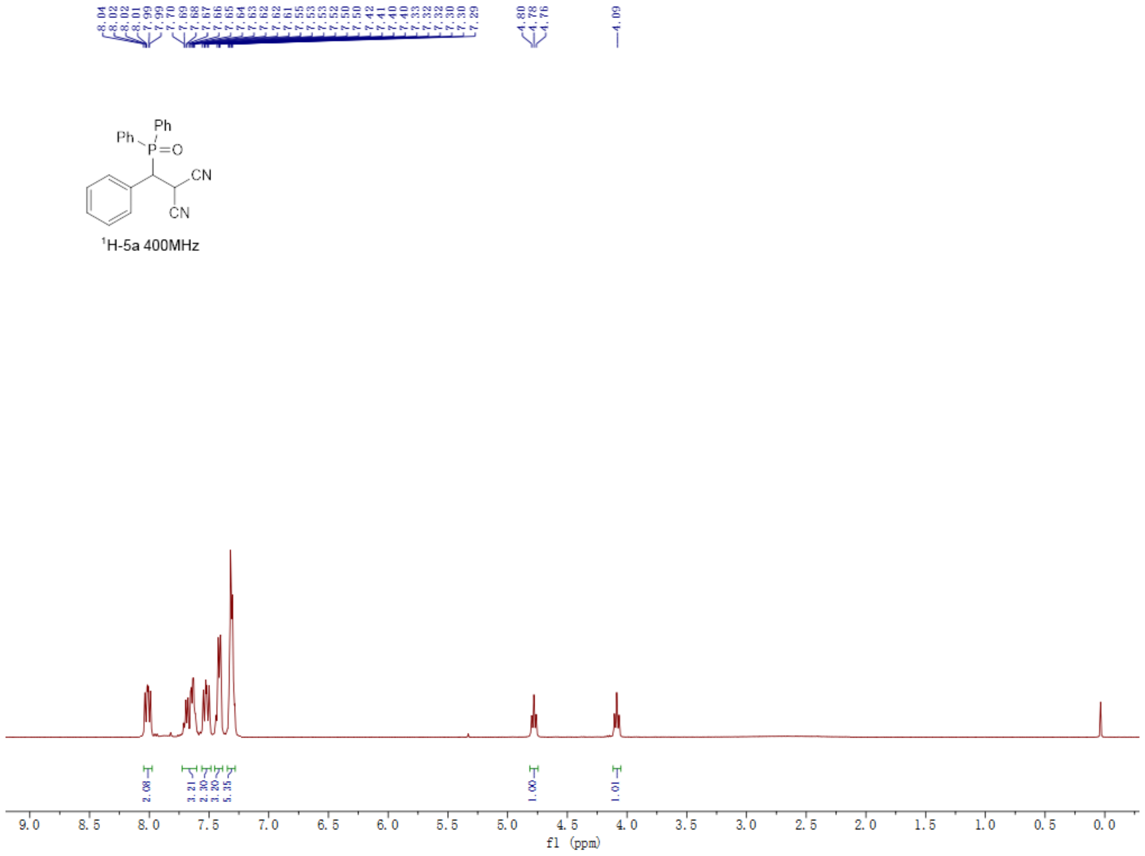
8.04 8.02 8.01 7.99 7.70 7.69 7.68 7.67 7.66 7.65 7.64 7.63 7.62 7.61 7.55 7.53 7.53 7.52 7.50 7.50 7.42 7.41 7.40 7.40 7.33 7.32 7.32 7.30 7.30 7.29
4.80 4.78 4.76
4.09
Ph
Ph
P=O
CN
CN
1H-5a 400MHz
2.08
3.21 2.20 2.20 5.35
1.00
1.01
9.0 8.5 8.0 7.5 7.0 6.5 6.0 5.5 5.0 4.5 4.0 3.5 3.0 2.5 2.0 1.5 1.0 0.5 0.0
f1 (ppm)

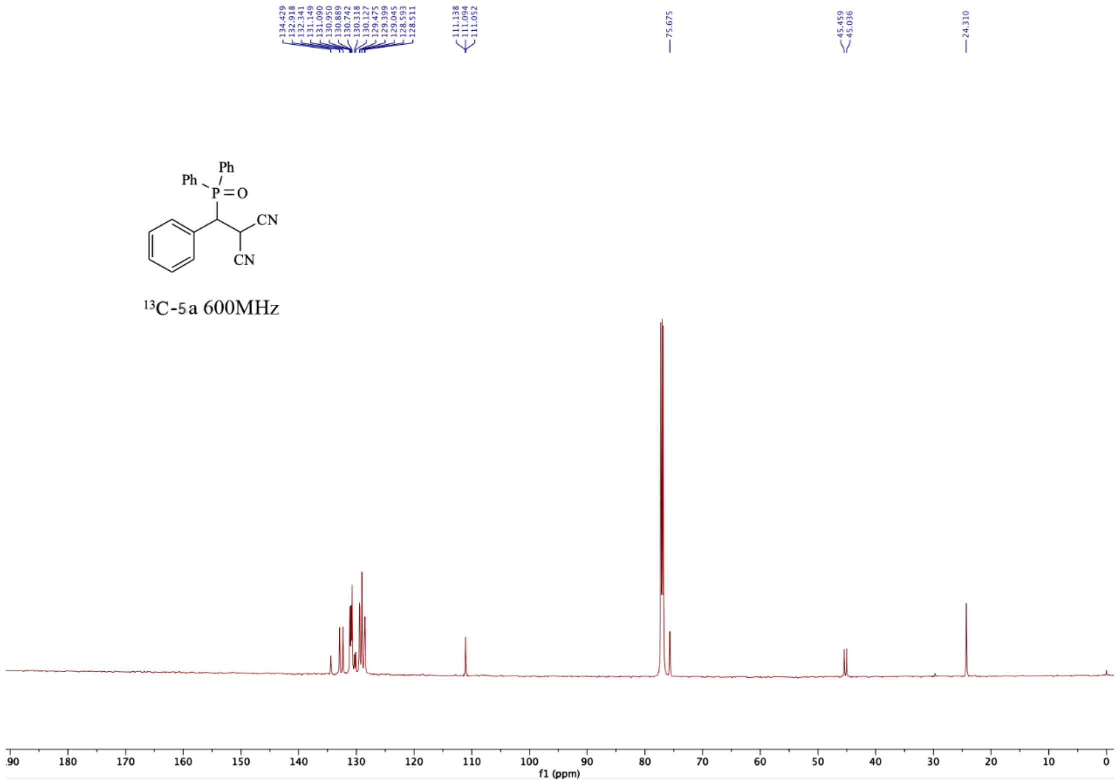

134.429
132.918
132.341
131.149
131.090
130.950
130.889
130.742
130.318
130.127
129.475
129.399
129.045
128.593
128.511
111.138
111.094
111.052
75.675
45.459
45.036
24.310
Ph
Ph
P=O
CN
CN
^{13}C-5a 600MHz
f1 (ppm)

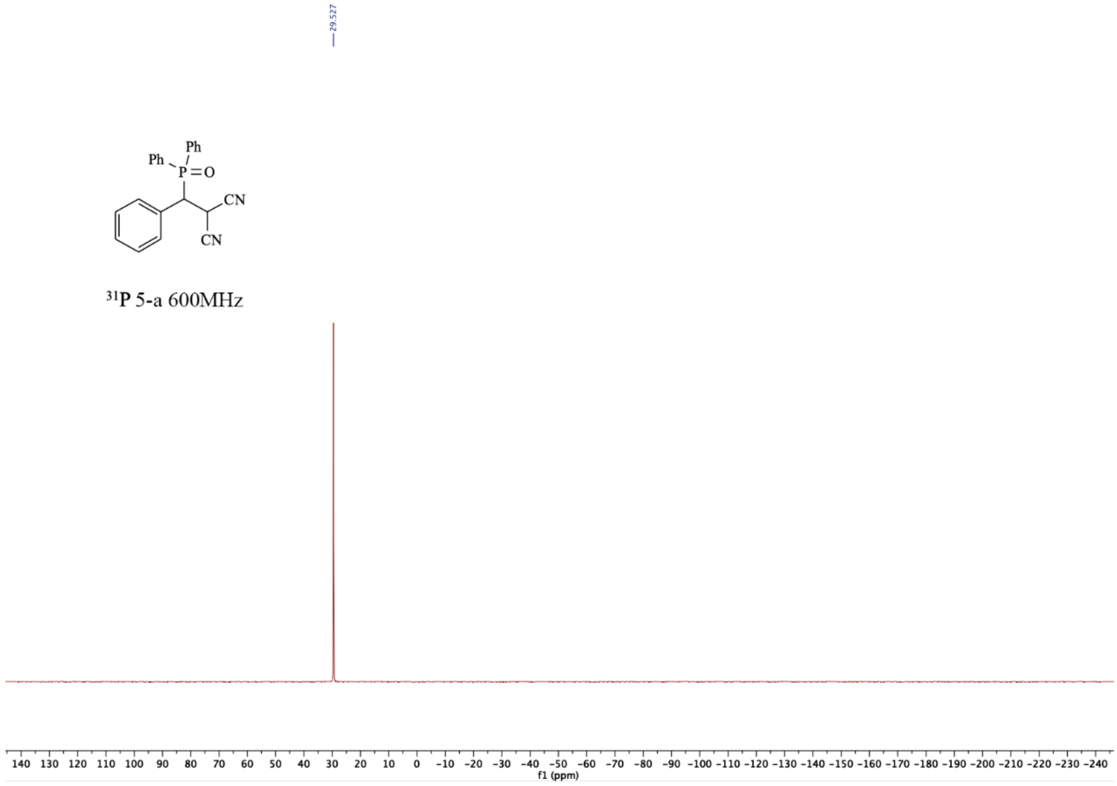

29.527
Ph
Ph
P=O
CN
CN
^{31}P 5-a 600MHz
f1 (ppm)

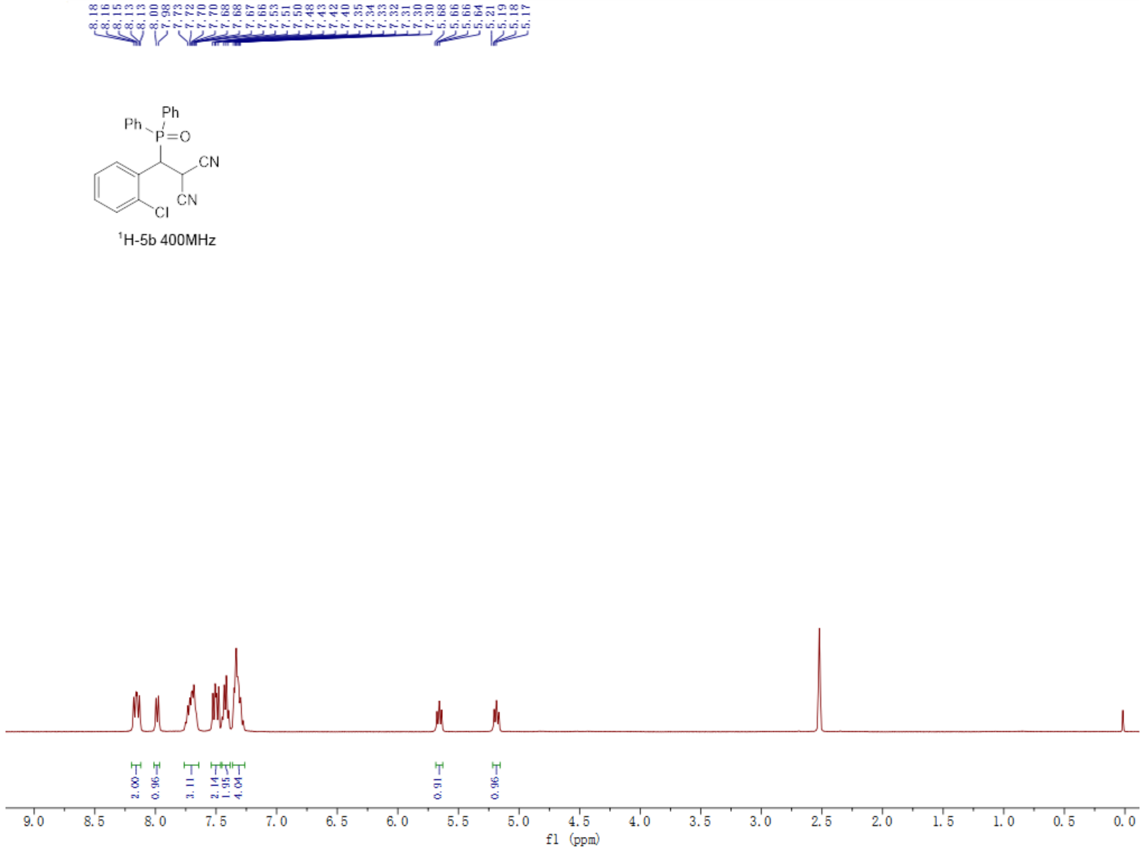
8.18
8.16
8.15
8.13
8.13
8.00
7.98
7.73
7.72
7.70
7.70
7.68
7.68
7.67
7.66
7.53
7.51
7.50
7.48
7.43
7.42
7.40
7.35
7.34
7.33
7.32
7.31
7.30
7.30
5.68
5.66
5.66
5.64
5.21
5.19
5.18
5.17
Ph
Ph
P=O
CN
CN
Cl
¹H-5b 400MHz
2.00
0.96
3.11
2.14
1.95
4.04
0.91
0.96
9.0
8.5
8.0
7.5
7.0
6.5
6.0
5.5
5.0
4.5
4.0
3.5
3.0
2.5
2.0
1.5
1.0
0.5
0.0
f1 (ppm)

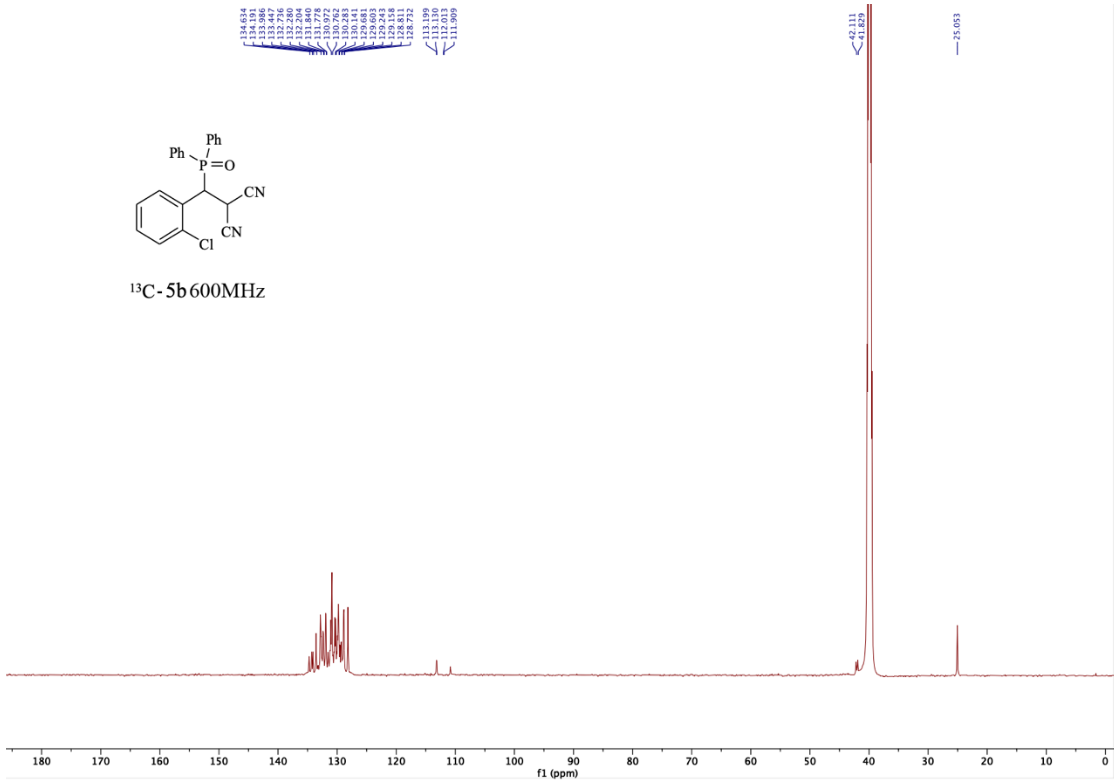
134.634
134.191
133.986
133.447
132.736
132.280
132.204
131.840
131.778
130.972
130.762
130.283
130.141
129.681
129.603
129.243
129.158
128.811
128.732
113.199
113.130
112.013
111.909
42.111
41.829
25.053
Ph
Ph
P=O
CN
CN
Cl
¹³C-5b 600MHz
180
170
160
150
140
130
120
110
100
90
80
70
60
50
40
30
20
10
0
f1 (ppm)

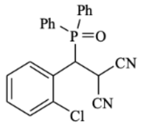

^{31}P 5-b 600MHz

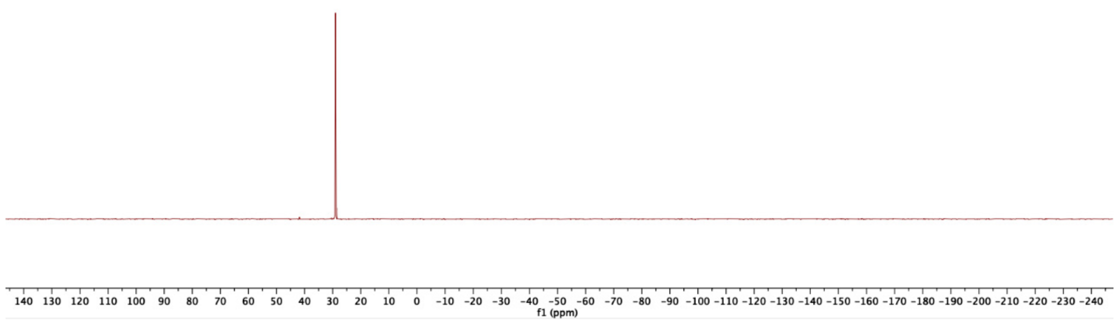

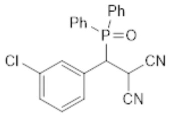

^{1}H-5c 400MHz

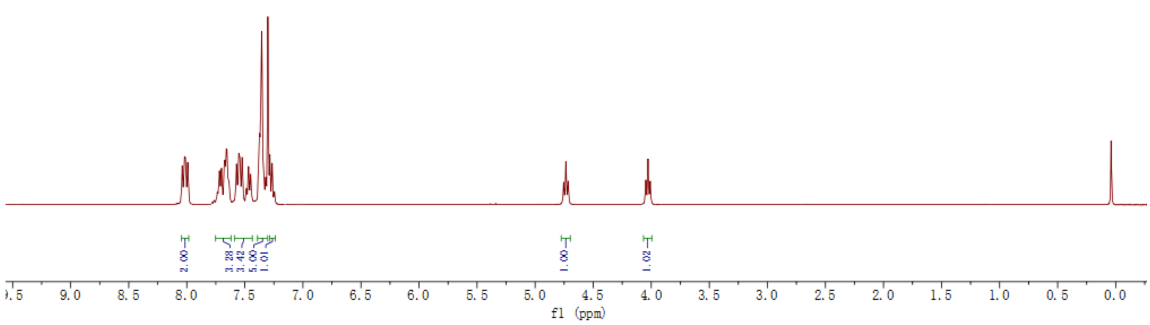

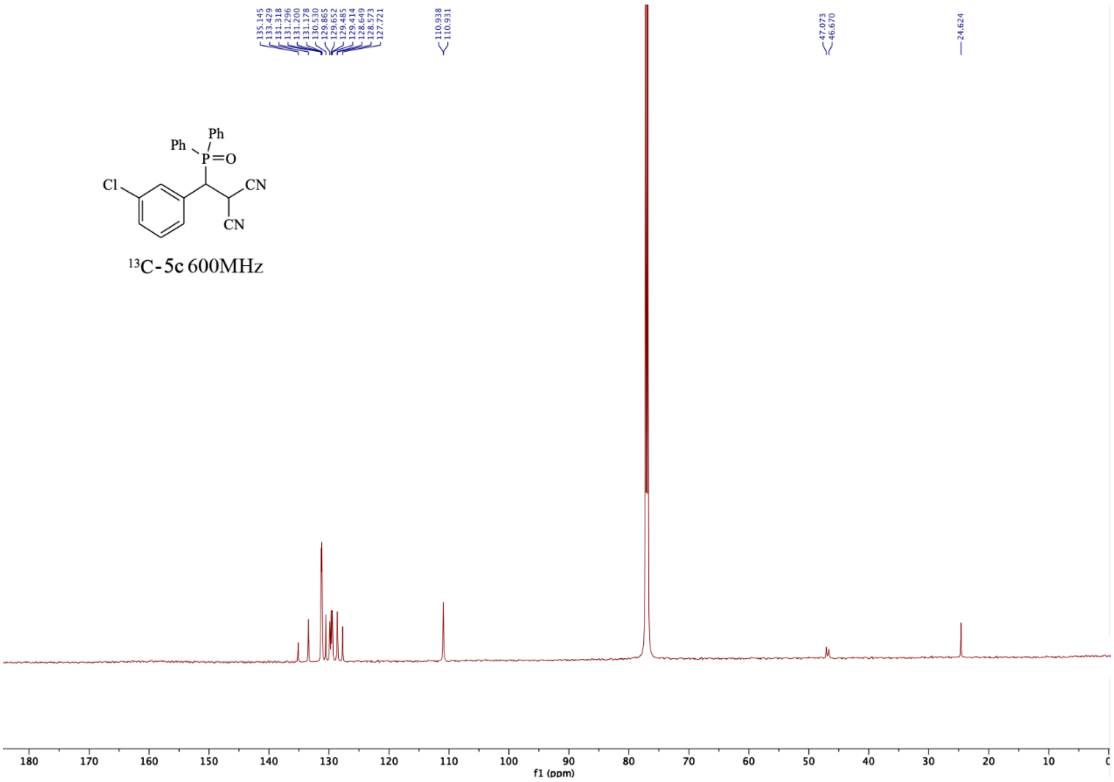

135.145
133.429
131.318
131.296
131.200
131.178
130.530
129.865
129.652
129.485
129.414
128.649
128.573
127.721
110.938
110.931
47.073
46.670
24.624
Ph
Ph
P=O
Cl
CN
CN
^{13}C-5c 600MHz
f1 (ppm)

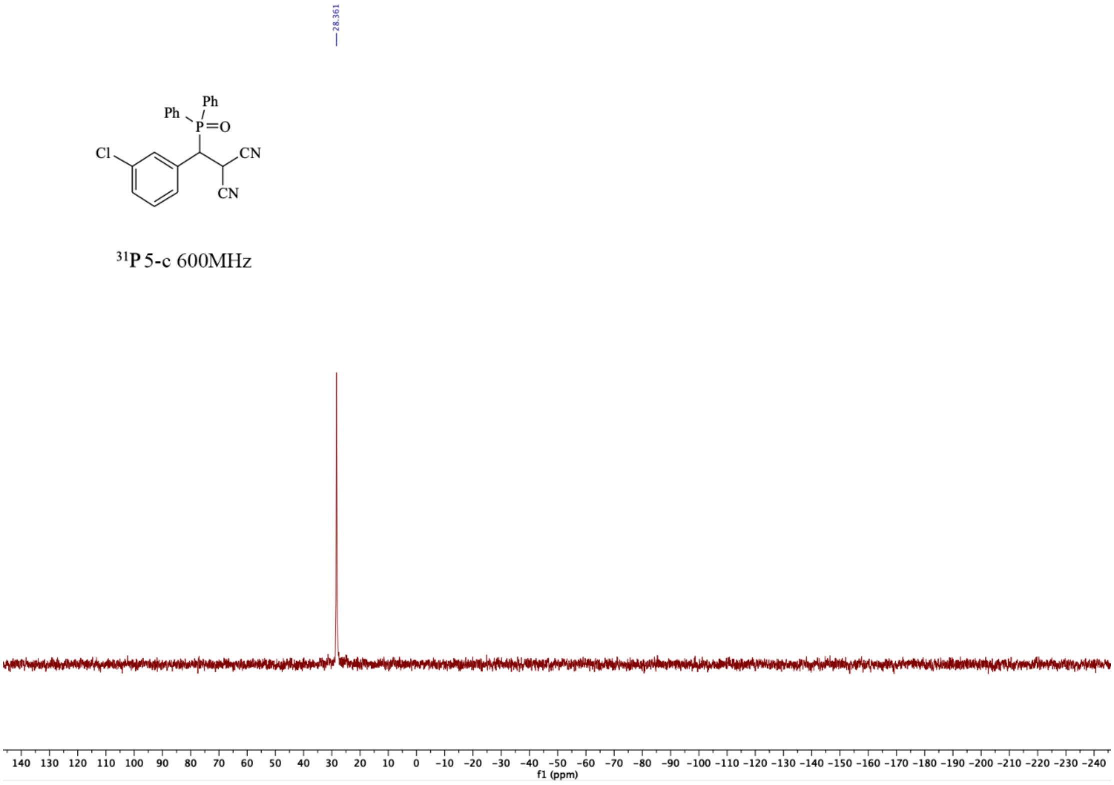

28.361
Ph
Ph
P=O
Cl
CN
CN
^{31}P 5-c 600MHz
f1 (ppm)

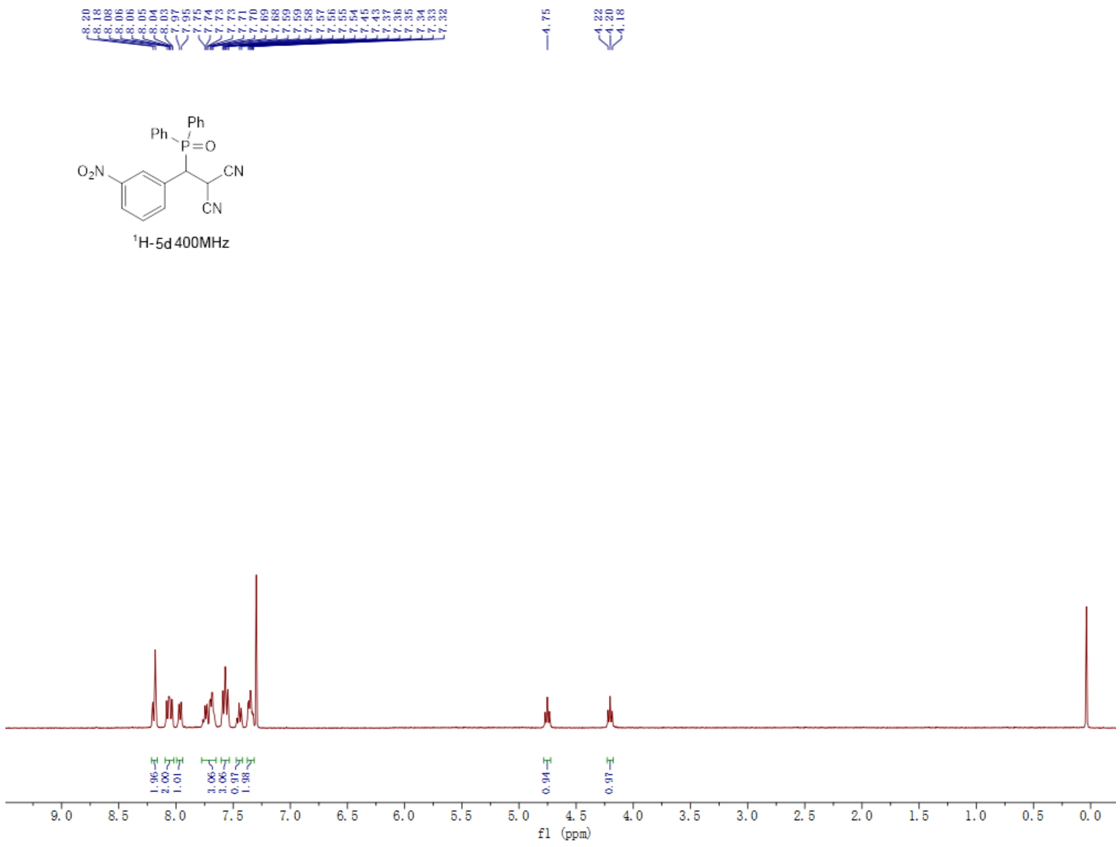
8.20 8.18 8.08 8.06 8.05 8.04 8.03 7.97 7.95 7.75 7.74 7.73 7.73 7.71 7.70 7.69 7.68 7.59 7.58 7.57 7.56 7.55 7.54 7.45 7.43 7.37 7.36 7.35 7.34 7.33 7.32
4.75
4.22 4.20 4.18
Ph
Ph
P=O
O2N
CN
CN
1H-5d 400MHz
1.96 2.00 1.01 3.06 3.06 0.97 1.98
0.94
0.97
9.0 8.5 8.0 7.5 7.0 6.5 6.0 5.5 5.0 4.5 4.0 3.5 3.0 2.5 2.0 1.5 1.0 0.5 0.0
f1 (ppm)

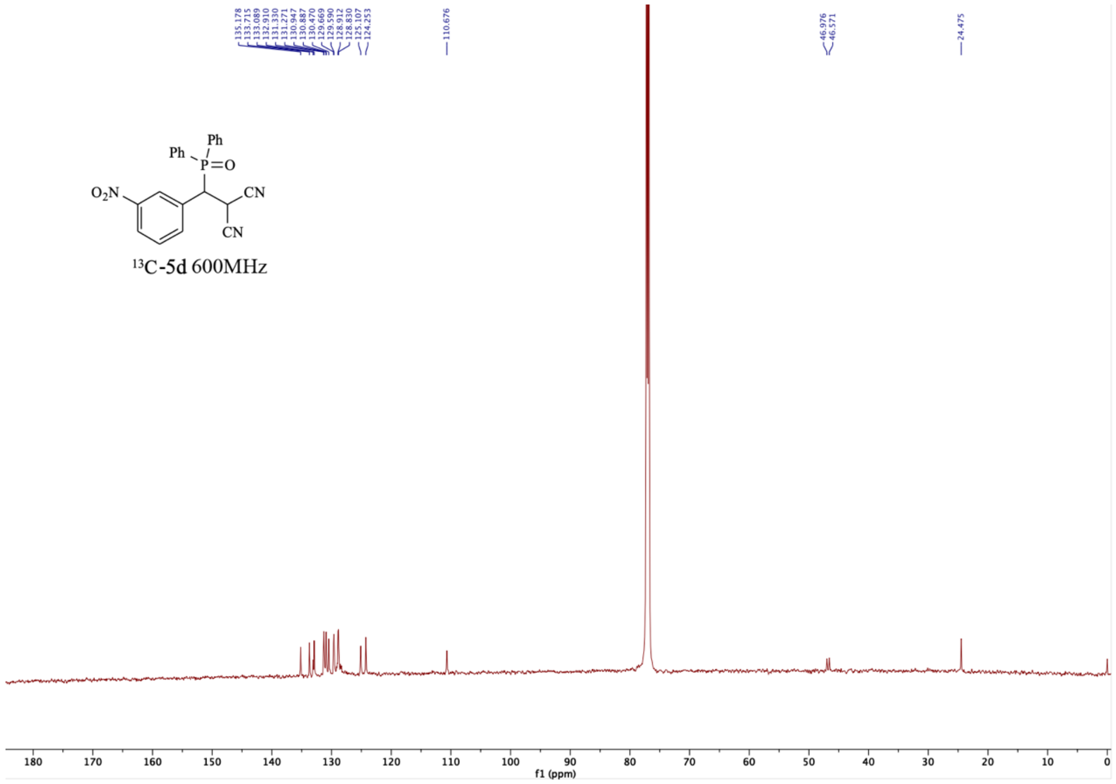
135.178 133.715 133.089 132.910 131.330 131.271 130.947 130.887 130.470 129.669 129.590 128.912 128.830 125.107 124.253
110.676
46.976 46.571
24.475
Ph
Ph
P=O
O2N
CN
CN
13C-5d 600MHz
180 170 160 150 140 130 120 110 100 90 80 70 60 50 40 30 20 10 0
f1 (ppm)

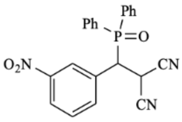

^{31}P 5-d 600MHz

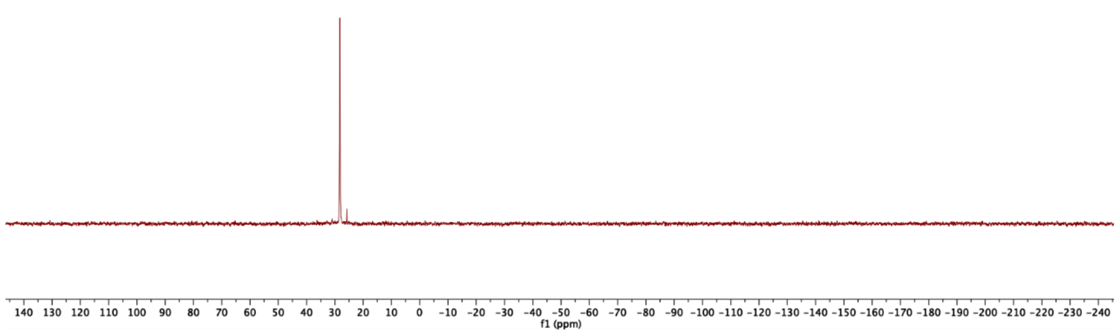

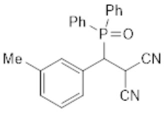

^{1}H-5e 400MHz

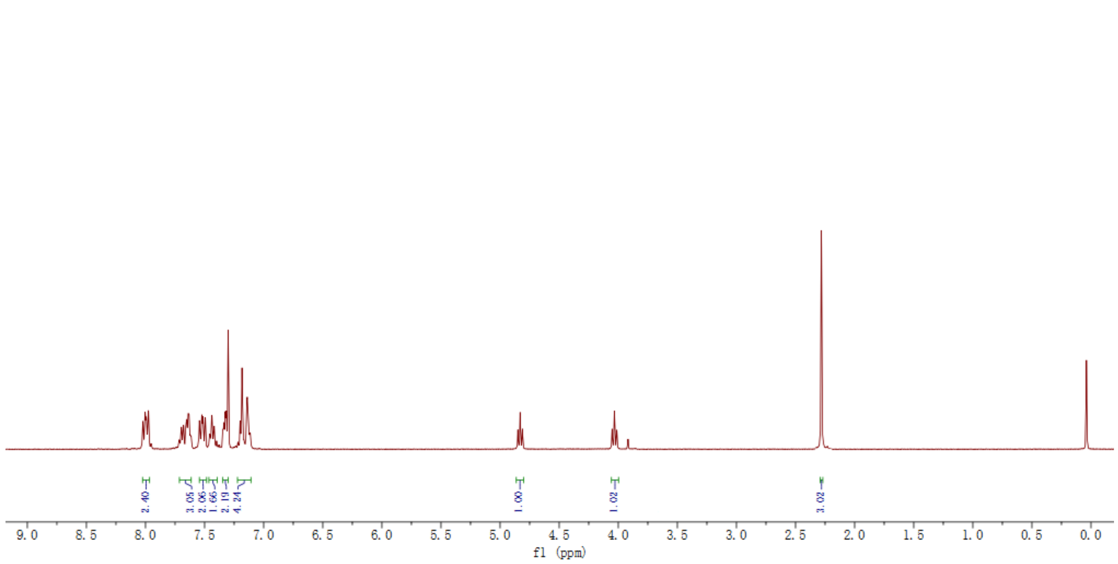

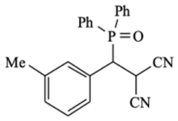

^{13}C-5e 600MHz

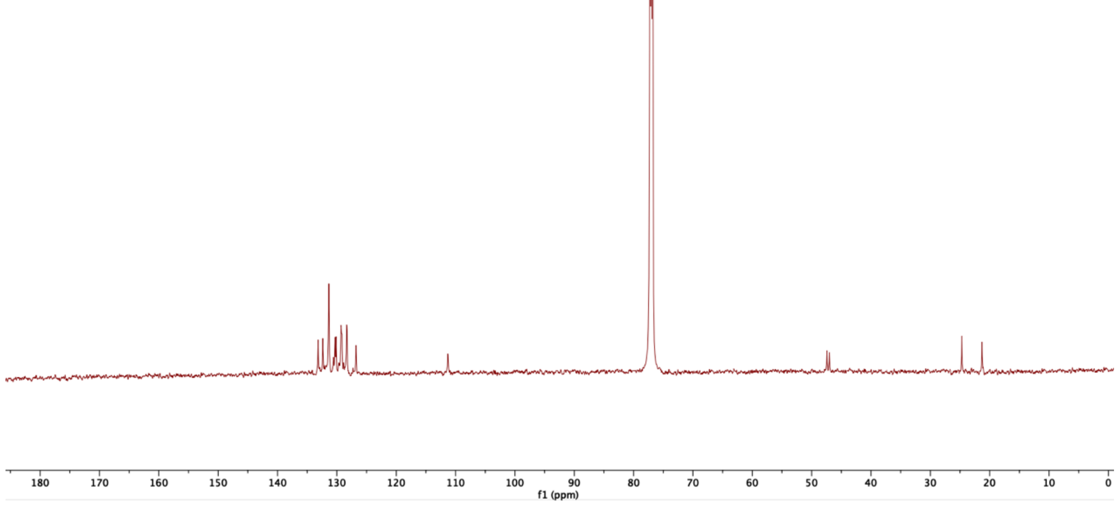

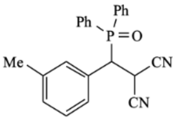

^{31}P 5-e 600MHz

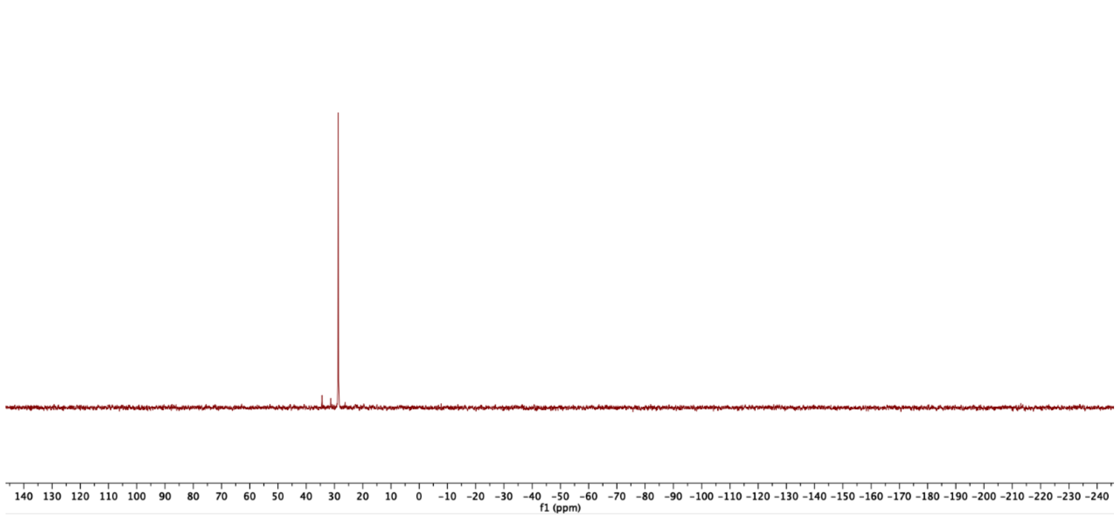

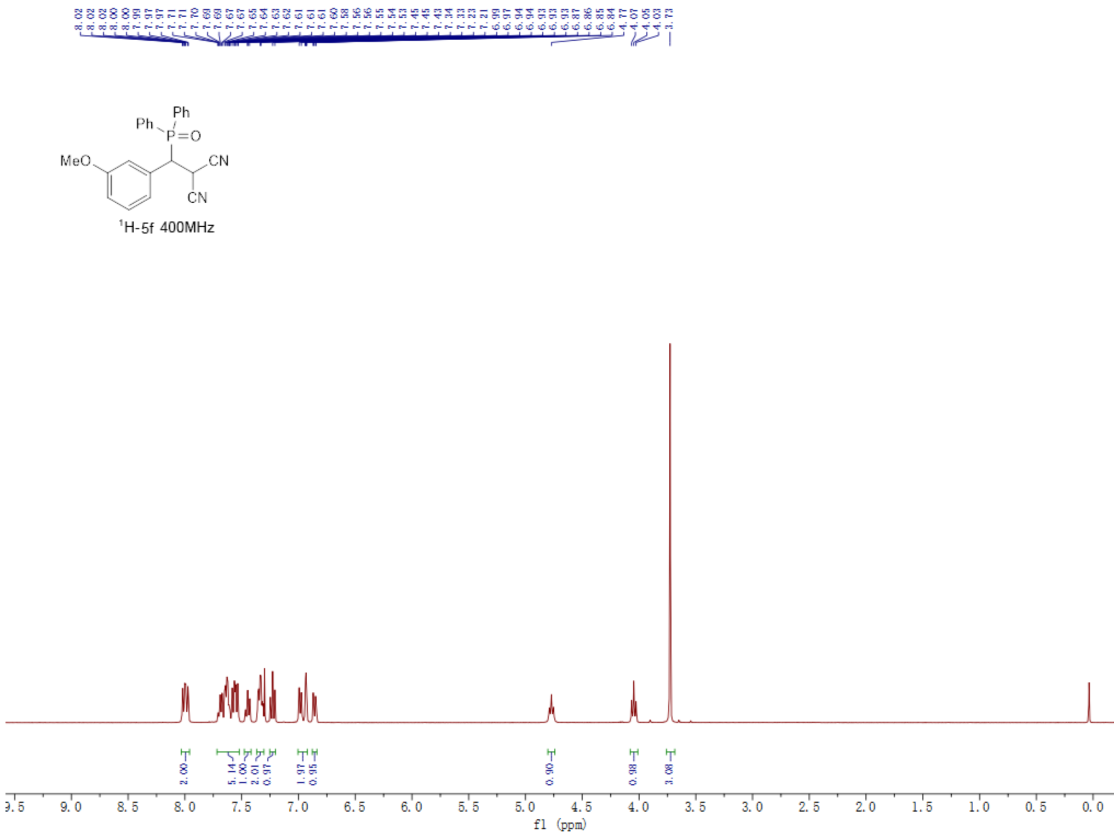
8.02 8.02 8.00 8.00 7.99 7.97 7.97 7.71 7.70 7.69 7.69 7.67 7.67 7.65 7.64 7.63 7.62 7.61 7.61 7.60 7.58 7.56 7.56 7.55 7.54 7.53 7.45 7.45 7.43 7.34 7.33 7.23 7.21 6.99 6.97 6.94 6.94 6.93 6.93 6.93 6.87 6.86 6.85 6.84 4.77 4.07 4.05 4.03 3.73
Ph
Ph
P=O
MeO
CN
CN
1H-5f 400MHz
2.00 5.14 1.00 2.01 0.97 1.97 0.95 0.90 0.98 3.08
9.5 9.0 8.5 8.0 7.5 7.0 6.5 6.0 5.5 5.0 4.5 4.0 3.5 3.0 2.5 2.0 1.5 1.0 0.5 0.0
f1 (ppm)

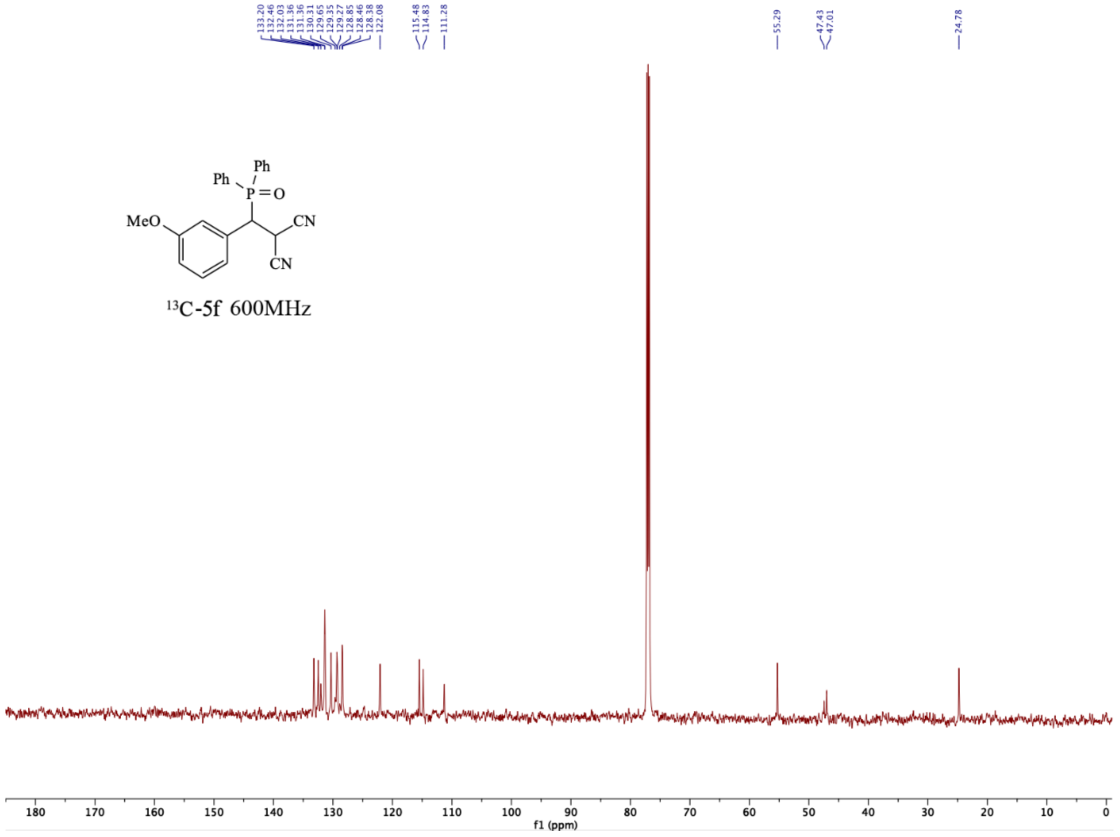
133.20 132.46 132.03 131.36 131.36 130.31 129.65 129.35 129.27 128.85 128.46 128.38 122.08 115.48 114.83 111.28 55.29 47.43 47.01 24.78
Ph
Ph
P=O
MeO
CN
CN
13C-5f 600MHz
180 170 160 150 140 130 120 110 100 90 80 70 60 50 40 30 20 10 0
f1 (ppm)

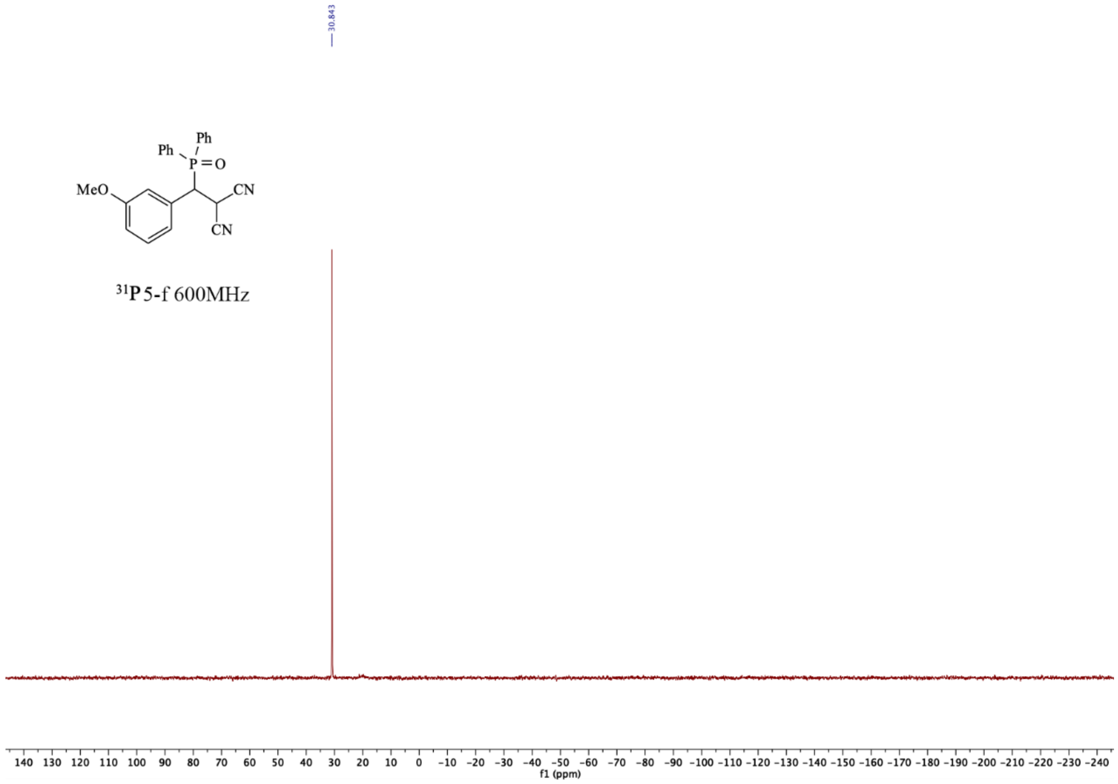

30.843
Ph
Ph
P=O
MeO
CN
CN
³¹P 5-f 600MHz
140 130 120 110 100 90 80 70 60 50 40 30 20 10 0 -10 -20 -30 -40 -50 -60 -70 -80 -90 -100 -110 -120 -130 -140 -150 -160 -170 -180 -190 -200 -210 -220 -230 -240
f1 (ppm)

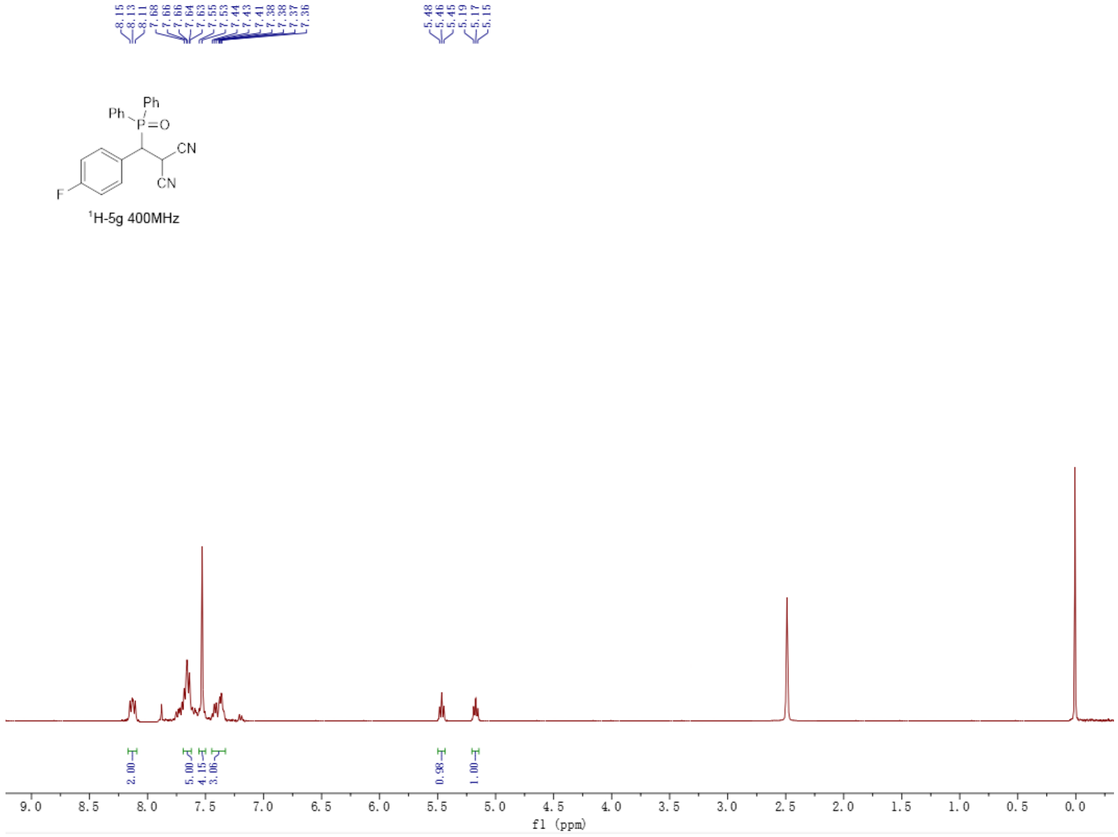

8.15 8.13 8.11 7.68 7.66 7.64 7.63 7.55 7.53 7.44 7.43 7.41 7.38 7.38 7.37 7.36
5.48 5.46 5.45 5.19 5.17 5.15
Ph
Ph
P=O
CN
CN
F
¹H-5g 400MHz
2.00
5.00 4.15 3.06
0.98
1.00
9.0 8.5 8.0 7.5 7.0 6.5 6.0 5.5 5.0 4.5 4.0 3.5 3.0 2.5 2.0 1.5 1.0 0.5 0.0
f1 (ppm)

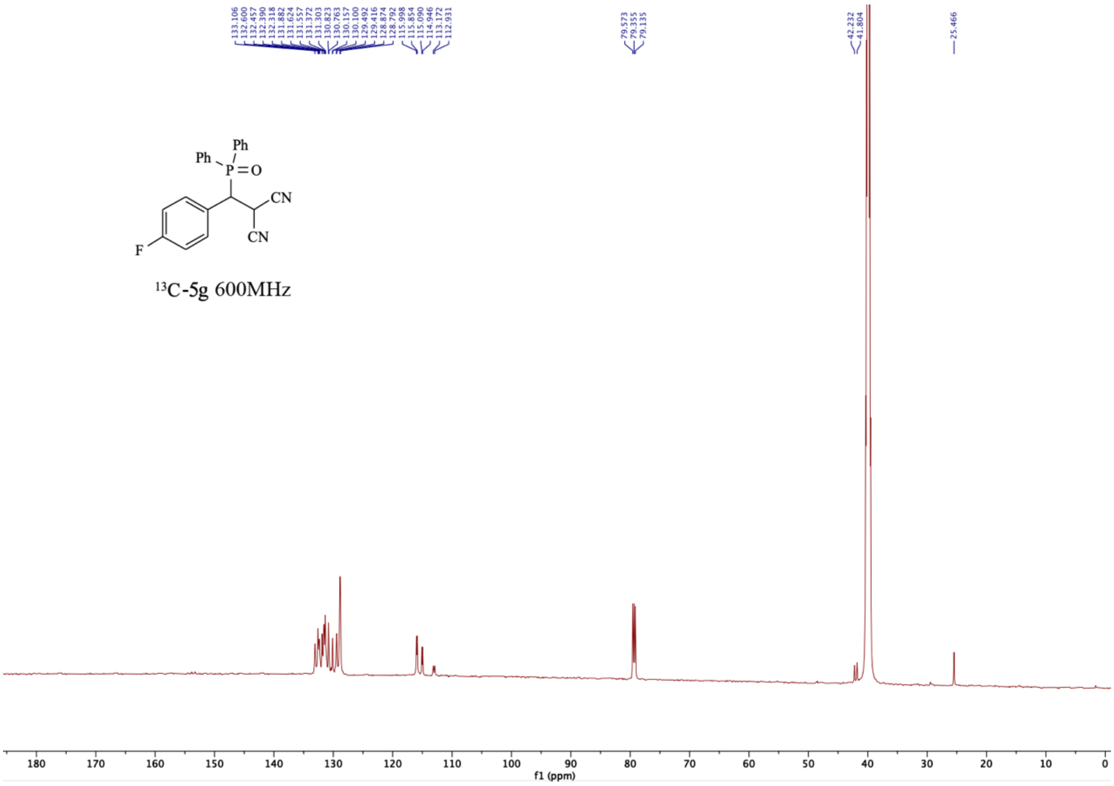

^{13}C-5g 600MHz

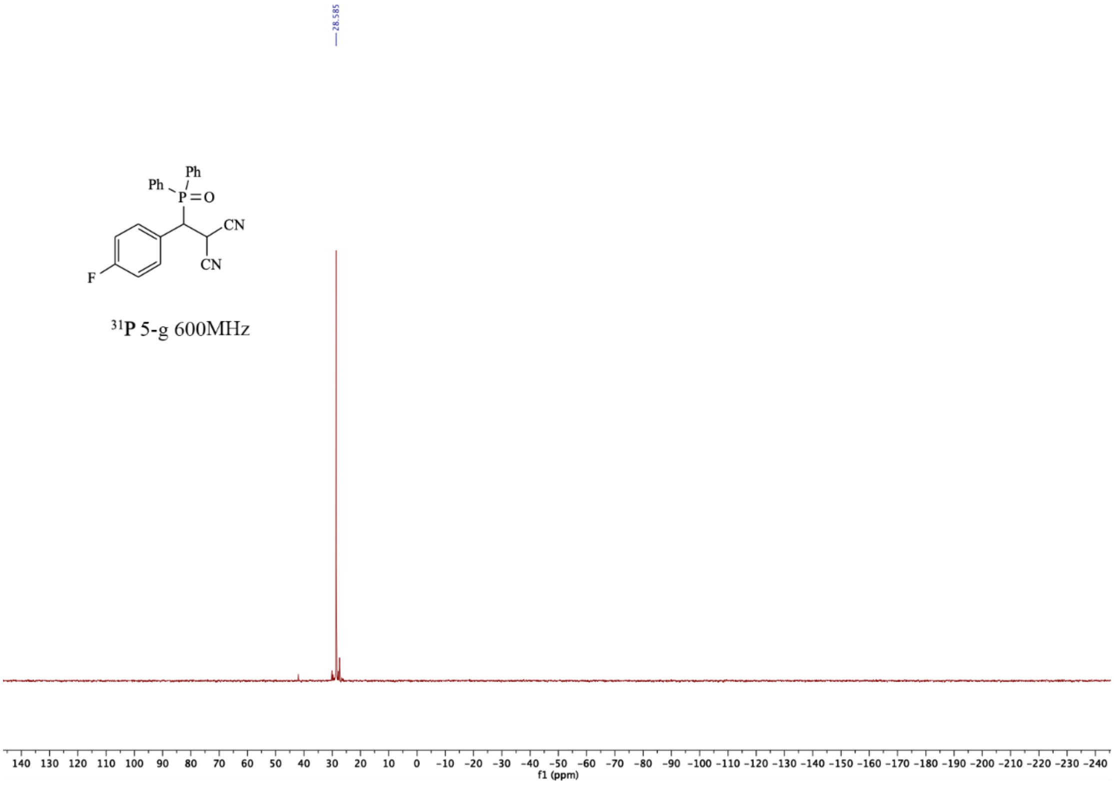

^{31}P 5-g 600MHz

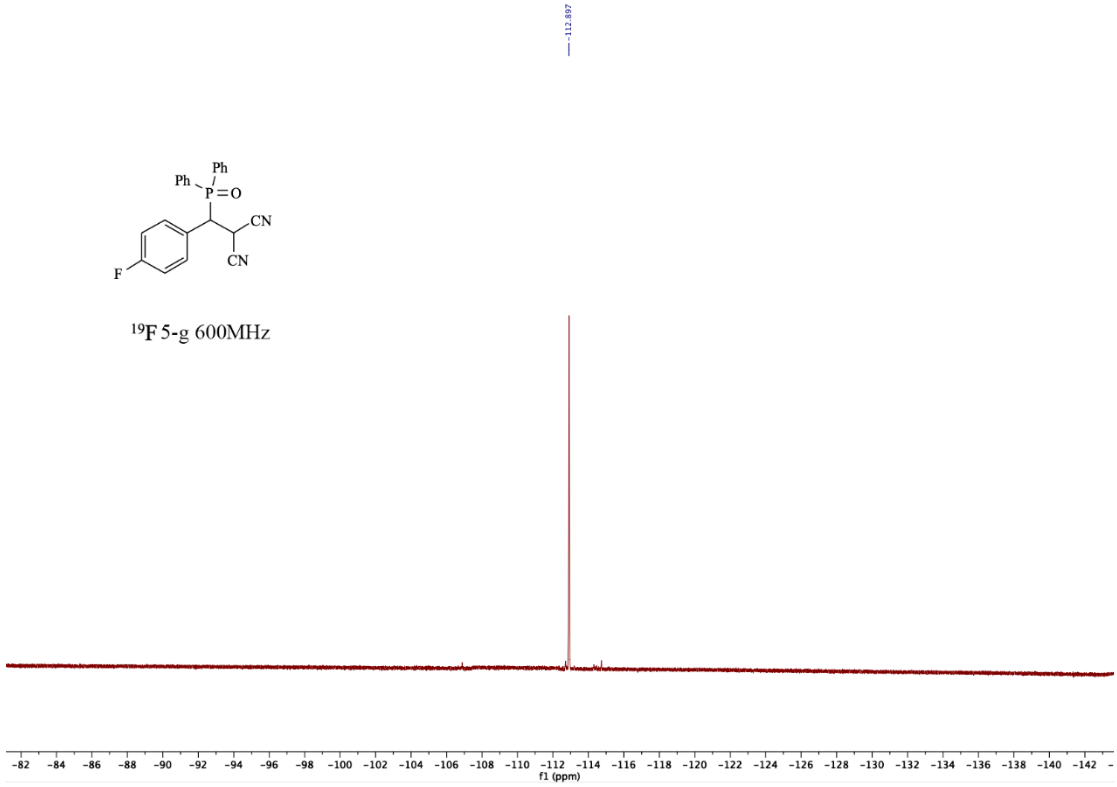
-112.897
Ph
Ph
P=O
CN
CN
F
19F 5-g 600MHz
f1 (ppm)

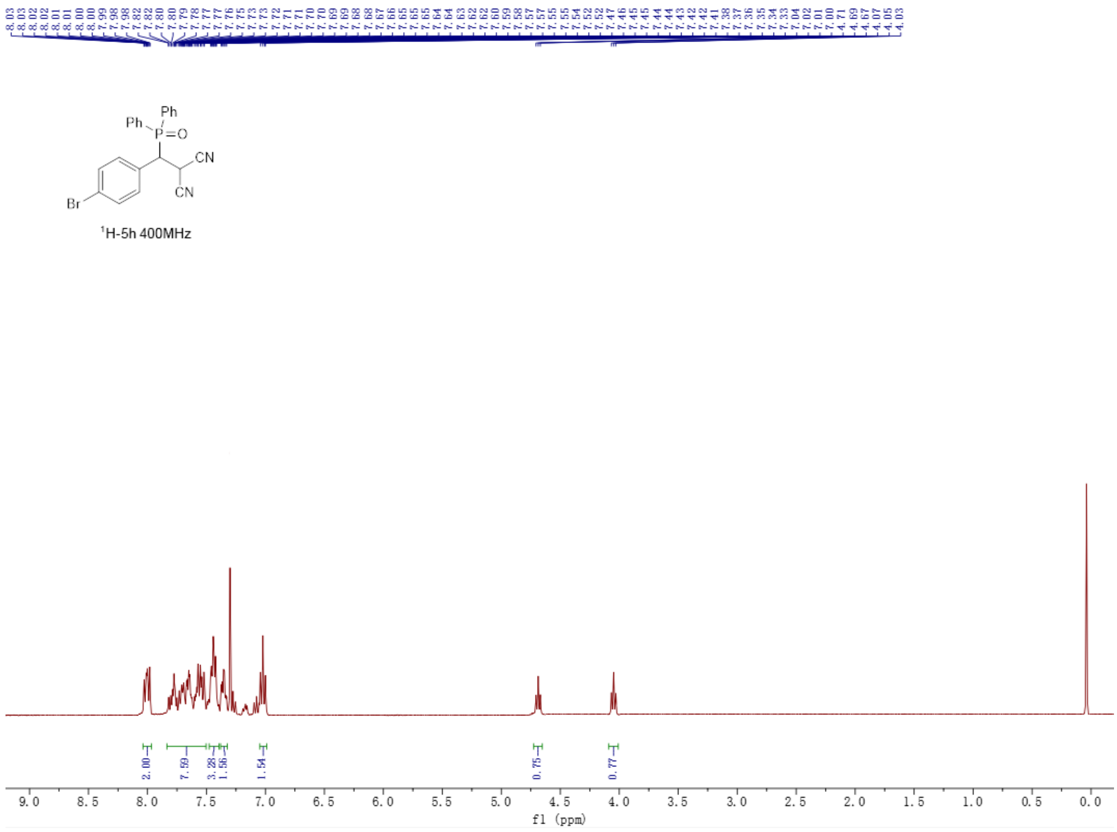
8.03 8.03 8.02 8.01 8.01 8.00 8.00 7.99 7.98 7.98 7.82 7.82 7.80 7.79 7.78 7.77 7.77 7.76 7.75 7.73 7.73 7.72 7.71 7.71 7.70 7.69 7.69 7.68 7.68 7.67 7.66 7.65 7.65 7.64 7.64 7.63 7.62 7.62 7.60 7.59 7.58 7.57 7.57 7.55 7.54 7.52 7.52 7.47 7.46 7.45 7.45 7.44 7.44 7.43 7.42 7.42 7.41 7.38 7.37 7.36 7.35 7.34 7.33 7.04 7.02 7.01 7.00 4.71 4.69 4.67 4.07 4.05 4.03
Ph
Ph
P=O
CN
CN
Br
1H-5h 400MHz
2.00
7.59
3.28
1.56
1.54
0.75
0.77
f1 (ppm)

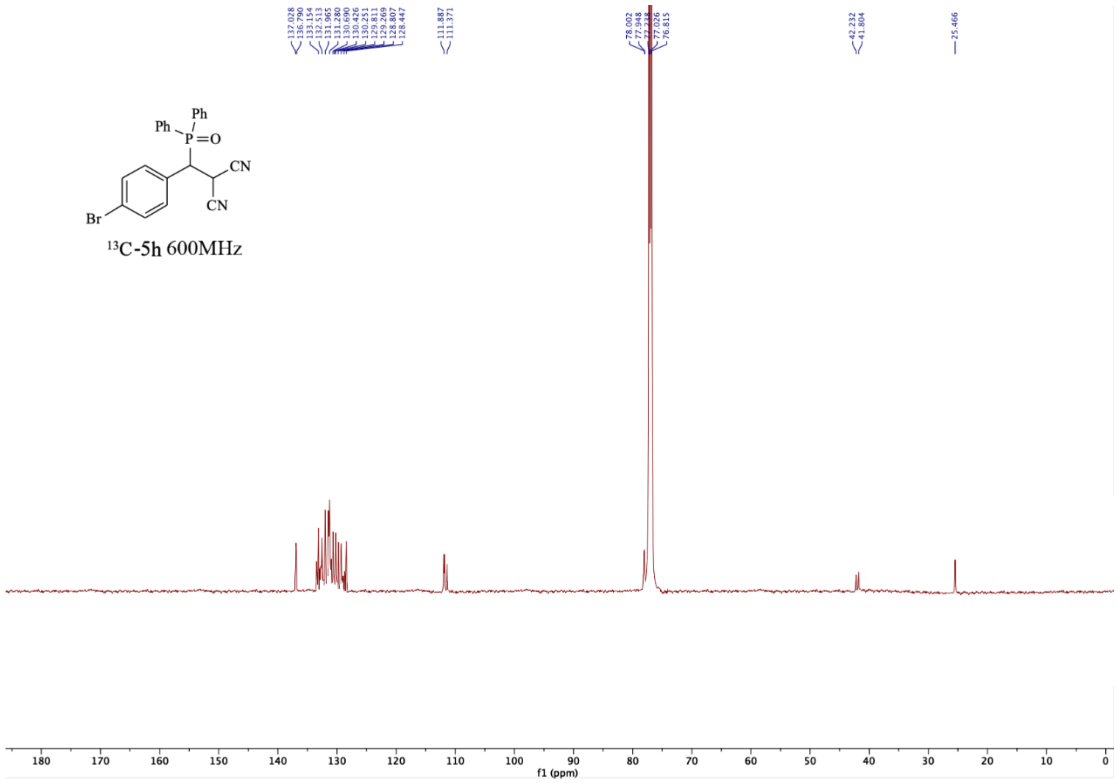

^{13}C-5h 600MHz
f1 (ppm)

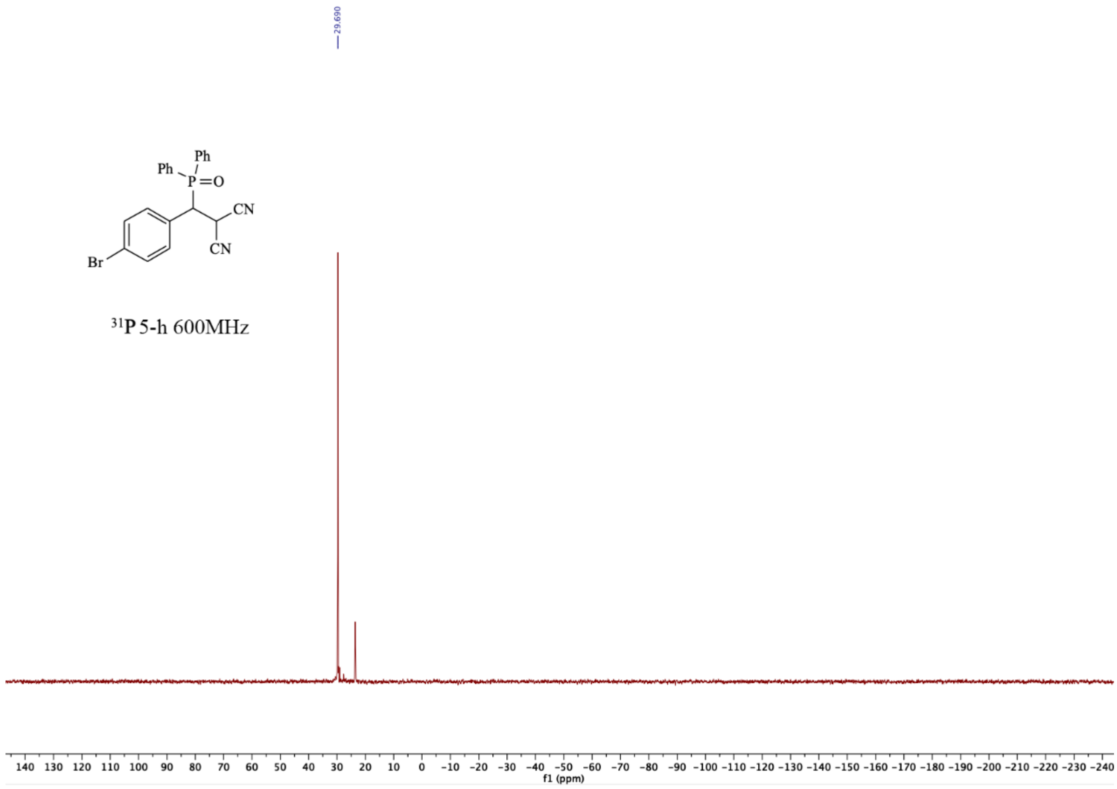

29.690
^{31}P 5-h 600MHz
f1 (ppm)

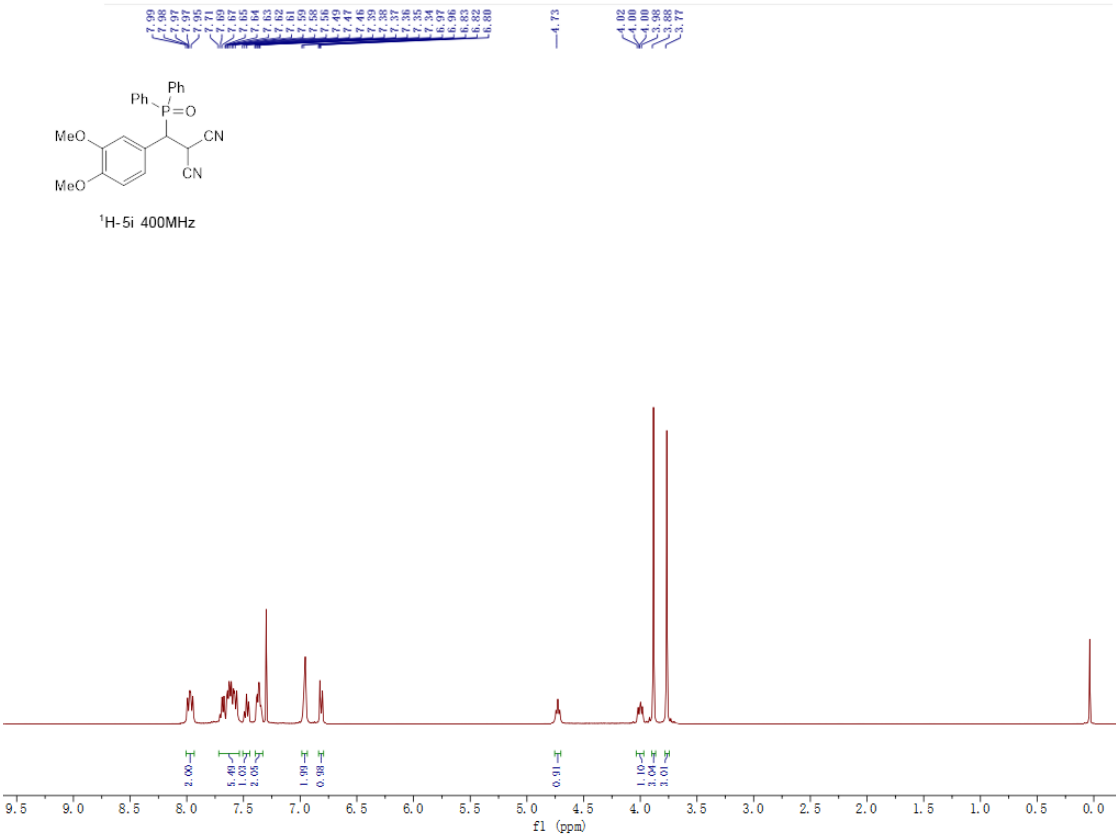

^{1}H-5i 400MHz

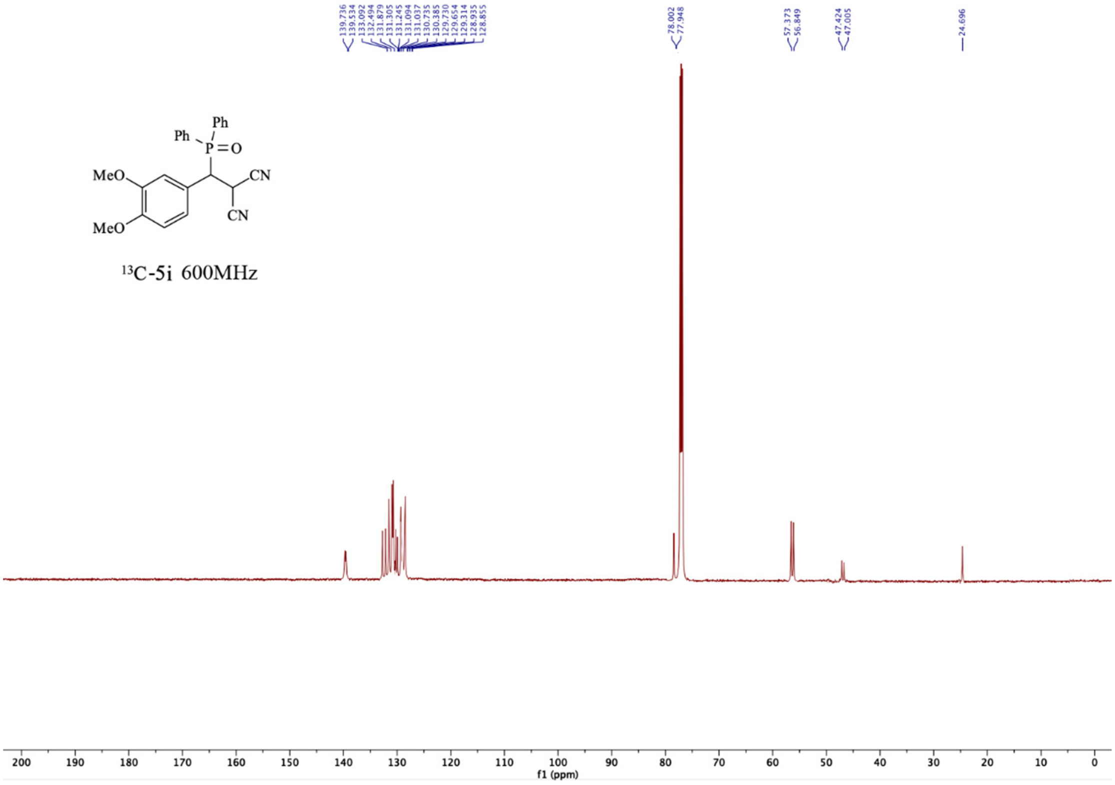

^{13}C-5i 600MHz

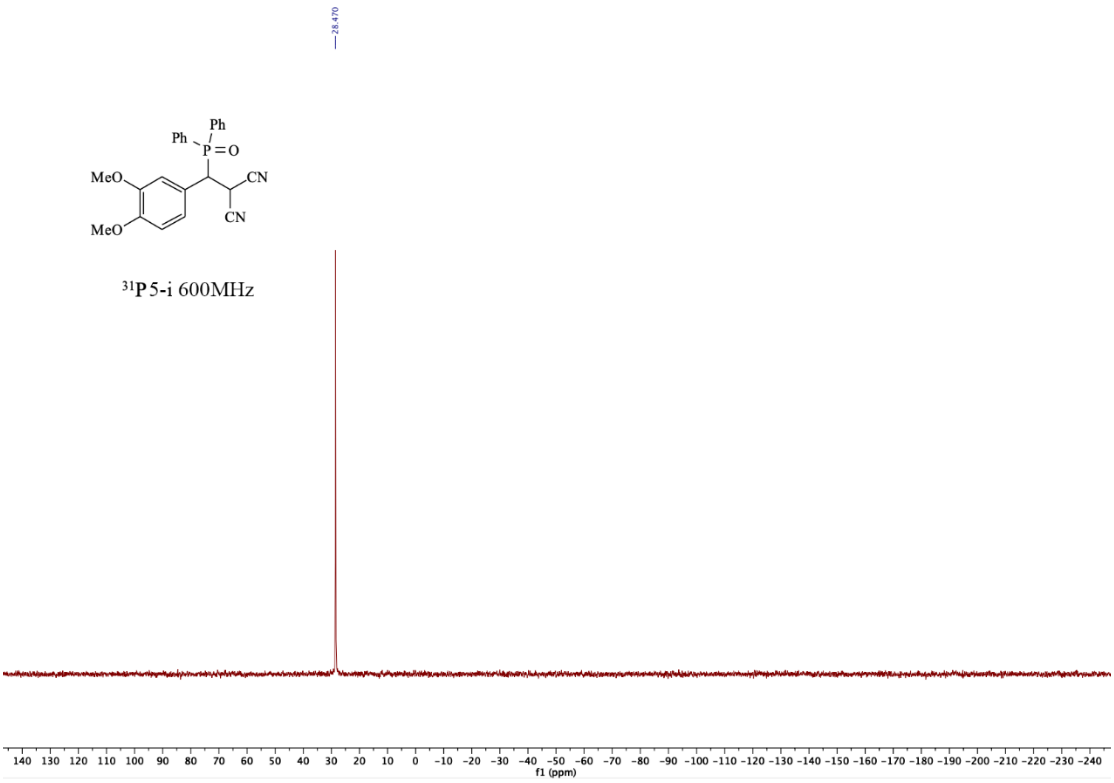

28.470
Ph
Ph
P=O
MeO
MeO
CN
CN
31P 5-i 600MHz
140 130 120 110 100 90 80 70 60 50 40 30 20 10 0 -10 -20 -30 -40 -50 -60 -70 -80 -90 -100 -110 -120 -130 -140 -150 -160 -170 -180 -190 -200 -210 -220 -230 -240
f1 (ppm)

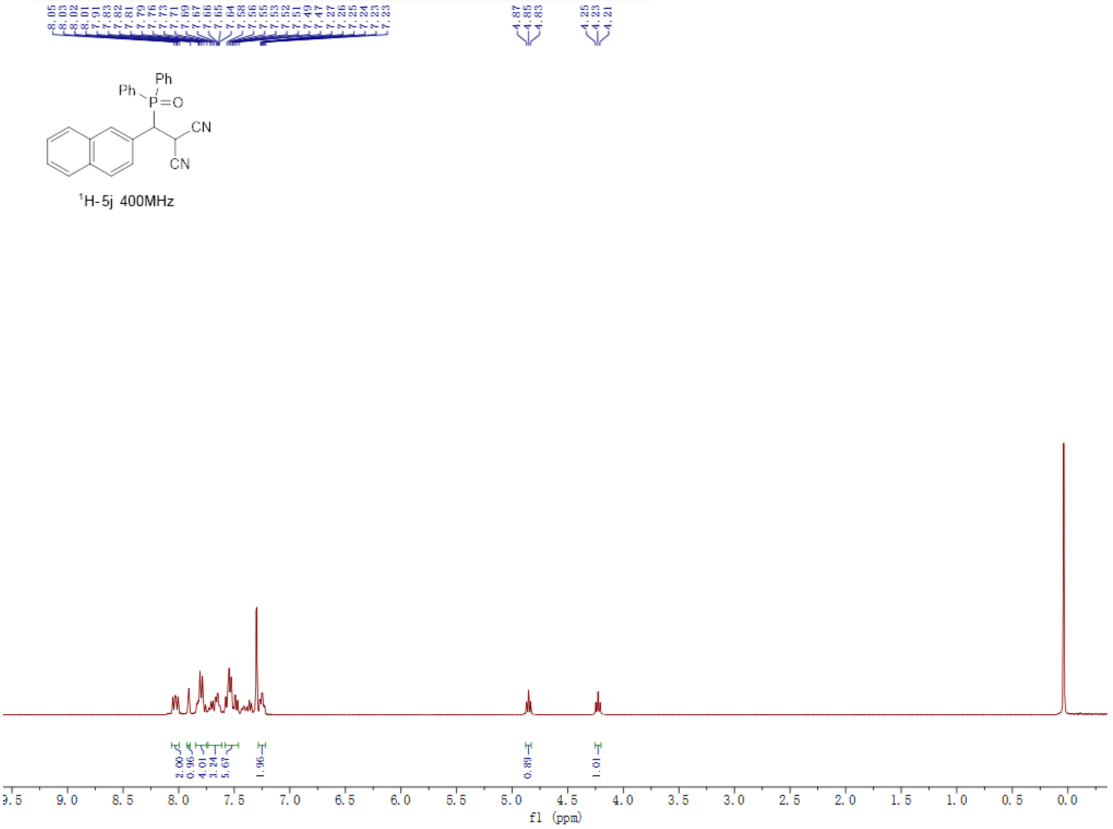

8.05 8.03 8.02 8.01 7.91 7.83 7.82 7.81 7.79 7.76 7.73 7.71 7.69 7.67 7.66 7.65 7.64 7.58 7.56 7.55 7.53 7.52 7.51 7.49 7.47 7.27 7.26 7.25 7.24 7.23 7.23
4.87 4.85 4.83
4.25 4.23 4.21
Ph
Ph
P=O
CN
CN
1H-5j 400MHz
2.00 0.96 4.01 3.24 5.67
1.96
0.89
1.01
9.5 9.0 8.5 8.0 7.5 7.0 6.5 6.0 5.5 5.0 4.5 4.0 3.5 3.0 2.5 2.0 1.5 1.0 0.5 0.0
f1 (ppm)

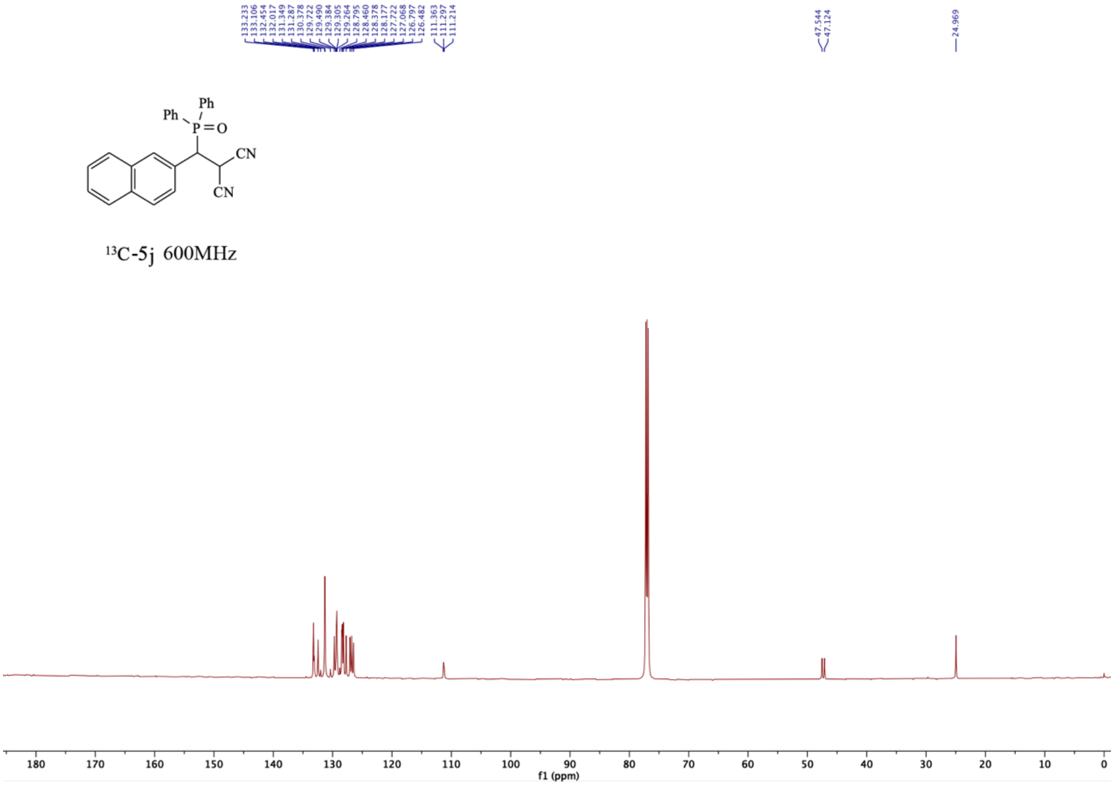
133.233
133.106
132.454
132.017
131.349
131.287
130.378
129.722
129.490
129.384
129.305
129.264
128.795
128.460
128.378
128.177
127.722
127.068
126.797
126.482
111.363
111.297
111.214
47.544
47.124
24.969
Ph
Ph
P=O
CN
CN
^{13}C-5j 600MHz
180 170 160 150 140 130 120 110 100 90 80 70 60 50 40 30 20 10 0
f1 (ppm)

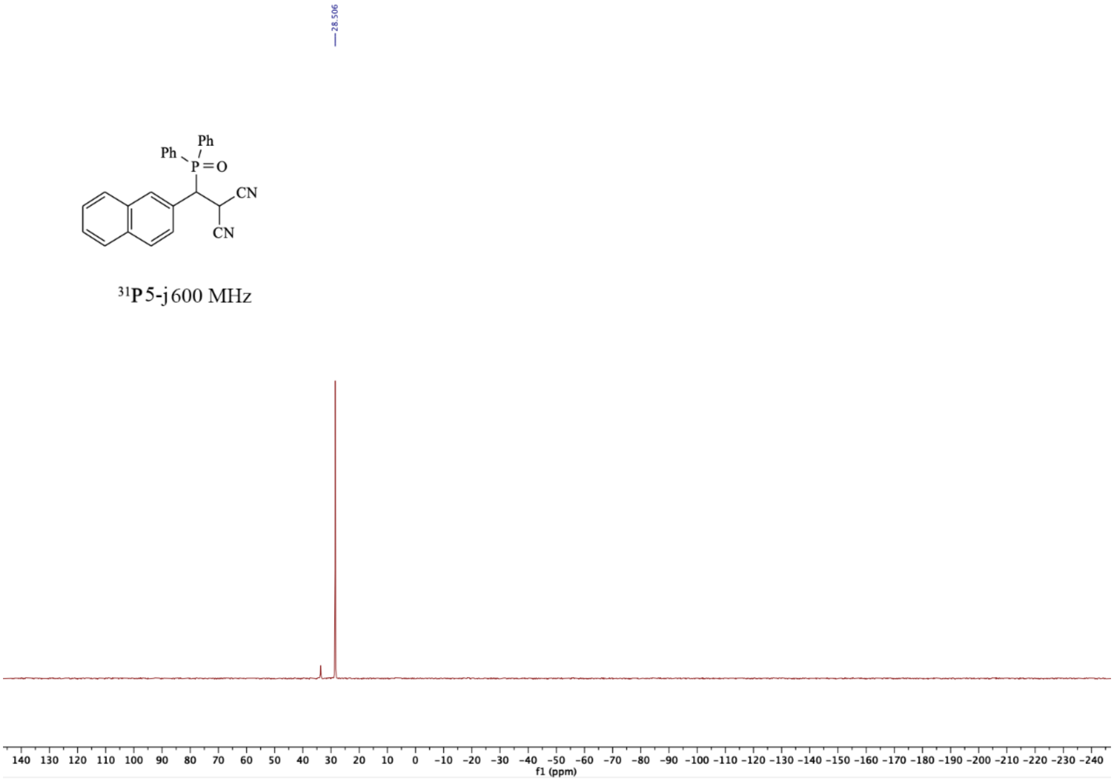
28.506
Ph
Ph
P=O
CN
CN
^{31}P 5-j 600 MHz
140 130 120 110 100 90 80 70 60 50 40 30 20 10 0 −10 −20 −30 −40 −50 −60 −70 −80 −90 −100 −110 −120 −130 −140 −150 −160 −170 −180 −190 −200 −210 −220 −230 −240
f1 (ppm)

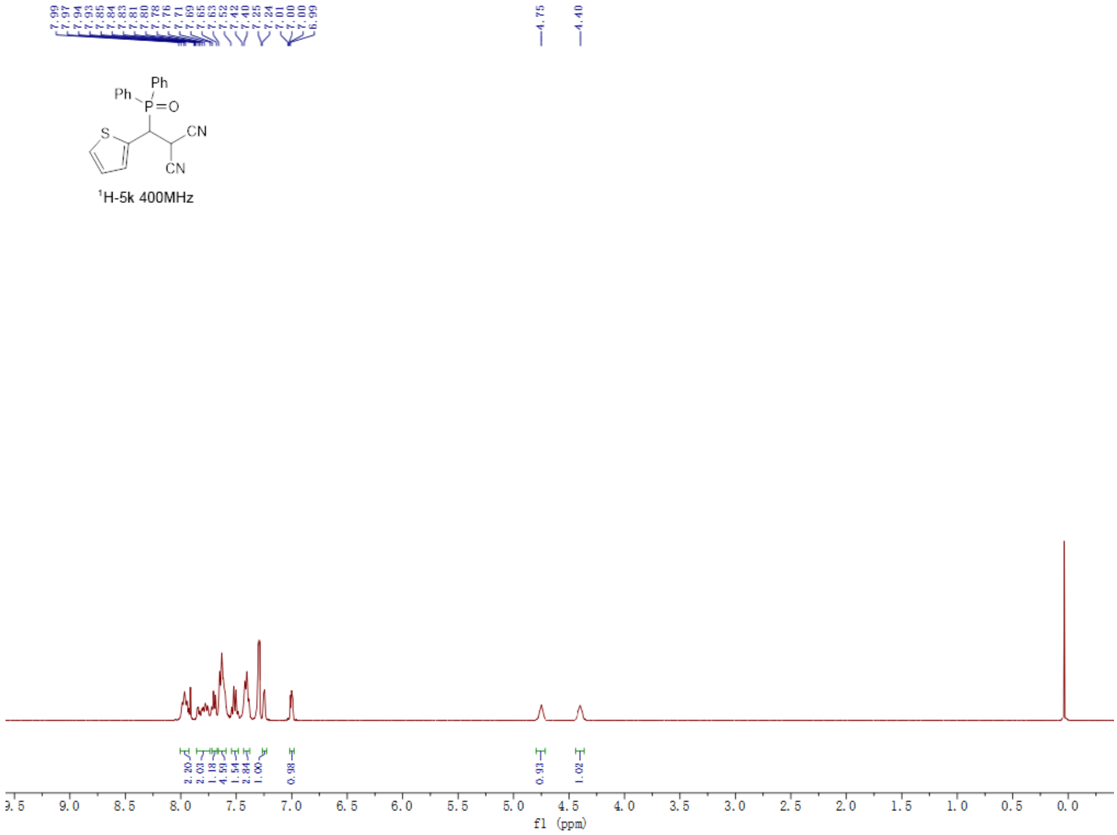

7.99
7.97
7.94
7.93
7.85
7.84
7.83
7.81
7.80
7.78
7.76
7.71
7.69
7.65
7.63
7.52
7.42
7.40
7.25
7.24
7.01
7.00
7.00
6.99
4.75
4.40
Ph
Ph
P=O
S
CN
CN
^{1}H-5k 400MHz
2.20
2.03
1.18
4.59
1.54
2.84
1.00
0.98
0.93
1.02
f1 (ppm)

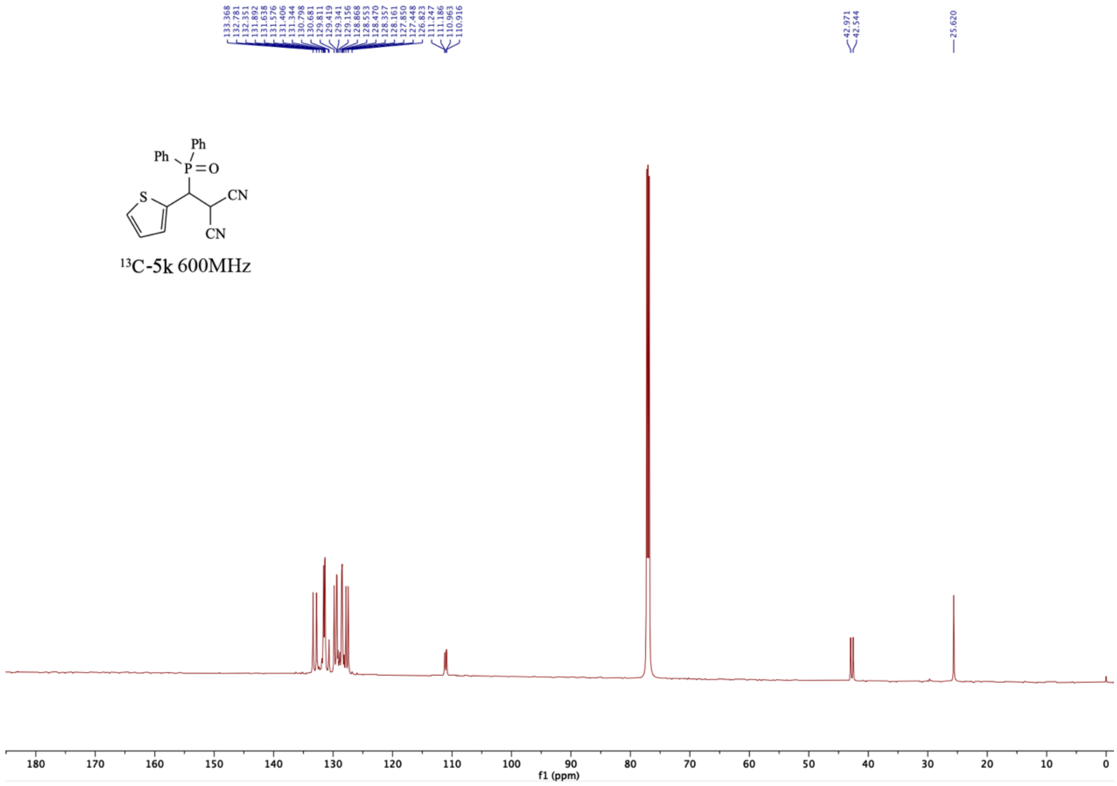

133.368
132.781
132.351
131.892
131.638
131.576
131.406
131.344
130.798
130.681
129.811
129.419
129.341
129.156
128.868
128.553
128.470
128.357
128.161
127.850
127.448
126.823
111.247
111.186
110.963
110.916
42.971
42.544
25.620
Ph
Ph
P=O
S
CN
CN
^{13}C-5k 600MHz
f1 (ppm)

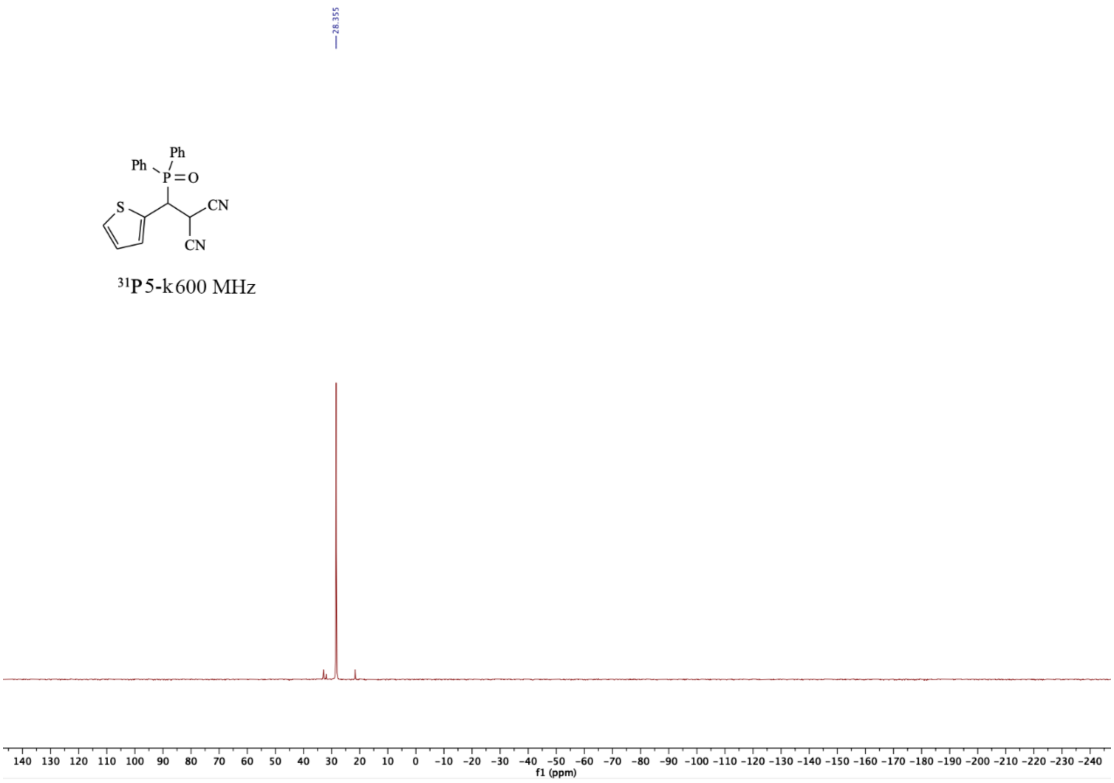
28.355
Ph
Ph
P=O
S
CN
CN
31P 5-k 600 MHz
140 130 120 110 100 90 80 70 60 50 40 30 20 10 0 -10 -20 -30 -40 -50 -60 -70 -80 -90 -100 -110 -120 -130 -140 -150 -160 -170 -180 -190 -200 -210 -220 -230 -240
f1 (ppm)
